# *Neurospora crassa* Δ*ccg-8* compromises cell surface integrity and antifungal tolerance: Insights from in vitro and *Galleria mellonella* studies

**DOI:** 10.1016/j.tcsw.2026.100172

**Published:** 2026-03-19

**Authors:** Ján Víglaš, Katarína Víglaš, Pavol Farkaš, Jana Bellová, Peter Baráth, Peter Gajdoš, Tatiana Klempová, Boris Lakatoš, Petra Olejníková

**Affiliations:** a Institute of Biochemistry and Microbiology, Faculty of Chemical and Food Technology, Slovak University of Technology in Bratislava, Radlinského 9, 81237 Bratislava, Slovakia; b Department of Nutrition and Food Quality Assessment, Faculty of Chemical and Food Technology, Slovak University of Technology in Bratislava, Radlinského 9, 81237 Bratislava, Slovakia; c Department of Glycobiotechnology, Institute of Chemistry, Slovak Academy of Sciences, Dúbravská cesta 9, 84538 Bratislava, Slovakia; d Department of Glycobiology, Institute of Chemistry, Slovak Academy of Sciences, Dúbravská cesta 9, 84538 Bratislava, Slovakia; e Institute of Biotechnology, Faculty of Chemical and Food Technology, Slovak University of Technology in Bratislava, Radlinského 9, 81237 Bratislava, Slovakia

**Keywords:** *Neurospora crassa*, Transcription factor *ccg-8*, *Galleria mellonella*, Proteins/glycans, Fatty acids

## Abstract

A comprehensive reference organism is often lacking when studying the adaptation of filamentous fungi to environmental stressors. This work investigates the mechanism underlying the response of *N. crassa* to stress conditions by focusing on the deletion mutant for the *CCG-8* transcription factor. Our molecular analyses revealed that Δ*ccg-8* severely compromises cell surface structure and metabolic homeostasis. Proteomic profiling demonstrated key dysregulations in ribosome biogenesis (consistent with the clock-controlled nature of *CCG-8*) and fatty acid *β*-oxidation. These findings, substantiated by changes in ergosterol and fatty acid composition, confirmed the increased susceptibility of deletion mutant to azoles and echinocandins. Furthermore, glycomic and proteomic data suggested that conidia of *N. crassa* Δ*ccg-8* exhibit protein and glycan alterations. To validate this structural compromise in vivo, we successfully applied the *Galleria mellonella* larval model. Despite being non-pathogenic, conidia of *N. crassa* Δ*ccg-8* were cleared significantly faster by the larval immune system than the wild-type strain, mirroring the in vitro observations. This work provides detailed molecular insights into fungal stress adaptation and establishes *N. crassa* as a viable non-pathogenic organism for in vivo analysis. This approach substantially broadens application of this filamentous fungus, enabling direct comparative research with pathogenic filamentous fungi in the domain of antifungal resistance and host interaction.

## Highlights

• The cell surface of *N. crassa* is substantially altered in the Δ*ccg-8* (*clock-controlled gene 8*) deletion mutant.
• The Δ*ccg-8* deletion modulates the susceptibility of *N. crassa* to various stress-causing agents, including antifungals.
• As a non-pathogenic model organism, *N. crassa* yields reproducible results in the in vivo model *Galleria mellonella.*

## 1 Introduction

In 2022, the World Health Organization (WHO) published its first list of priority fungal pathogens to elevate research on antifungal resistance (WHO, 2022). While resistance is robustly studied, a critical gap remains in understanding tolerance mechanisms, which often cause therapy failure. Unlike resistance, which is generally attributed to genomic variations, tolerance is a stress-induced reaction triggered by signaling pathways responding to antifungal agents. Macroscopically, tolerance appears as the ability of a fungus to grow in the presence of an antifungal drug at concentrations above the minimum inhibitory concentration (MIC) (Berman and Krysan, 2020; Robbins et al., 2017).

Understanding both resistance and tolerance often relies on the well-characterized (non-pathogenic) model organisms such as *Saccharomyces cerevisiae*. This is a logical choice, especially when studying high-priority yeast pathogens like *Cryptococcus neoformans*, *Candida auris*, and *Candida albicans*. However, this model is less suitable for the pathogen belonging to filamentous fungi, i.e., *Aspergillus fumigatus* [together with aforementioned yeast, a member of the highest priority pathogens for research (WHO, 2022)].

A more appropriate model for studying stress adaptation in filamentous fungi might be *Neurospora crassa*, a well-established fungus, characterized genetically and physiologically (Gu et al., 2016; Verdín et al., 2019). Although *N. crassa* is non-pathogenic, its study in the field of antifungal tolerance is justified because stress response pathways are evolutionarily conserved among eukaryotes. Crucially, *N. crassa* is phylogenetically closer to *A. fumigatus* (both subphylum *Pezizomycotina*) than *S. cerevisiae* (subphylum *Saccharomycotina*), providing a more relevant insight into adaptation processes (Víglaš and Olejníková, 2021). Tolerance mechanisms are, in fact, a core part of resistance studies, as a fungus must first withstand the pressure of agent to evolve resistance (Berman and Krysan, 2020).

*N. crassa* has been utilized to study antifungal stress response, leading to the description of several transcription factors (TFs) regulating azole susceptibility: *CSP-1*, *ADS-4*, *ADS-1*, and *CCG-8* (X. Chen et al., 2016; Wang et al., 2015; Xue et al., 2019; Yin et al., 2021). Bioinformatic studies suggested that TF *CCG-8* may be involved in a broader stress response (Hu et al., 2018).

In this work, we analyzed the impact of deleting the *ccg-8* gene on the ability of *N. crassa* to withstand various stress conditions in vitro and in vivo. Our analysis confirmed the broad role of *CCG-8*, as the deletion mutant *N. crassa* Δ*ccg-8* displayed increased susceptibility to various cell surface stressors, including antifungal agents. Furthermore, the Δ*ccg-8* mutation impacted the immune response of *Galleria mellonella* larvae – a model for in vivo studies of fungal infections caused by *Candida* spp., *Malassezia* spp., *Cryptococcus neoformans*, and *Aspergillus fumigatus*, as well as in investigations of potential treatments for these infections (Giammarino et al., 2024; Wojda et al., 2020). Differences in the cell wall and plasma membrane of the wild-type and Δ*ccg-8* strain were confirmed by glycomic and proteomic analyses. We showed that *CCG-8* coordinates stress networks beyond azole adaptation. This study highlights the value of *N. crassa* in stress tolerance research and provides arguments for novel avenues using non-pathogens for in vivo studies.

## 2 Materials and methods

### 2.1 Microbial strains and cultivation conditions

We assessed the role of the clock-controlled transcription factor *CCG-8* (encoded by *ccg-8*; NCU09686) using the wild-type strain *Neurospora crassa* FGSC2489 (mating type “A”, abbreviated as wtA) and the deletion mutant *N. crassa* Δ*ccg-8* FGSC20738. Both strains were originally generated as part of the *Neurospora* genome-wide knockout project (Colot et al., 2006), and were obtained from the Fungal Genetic Stock Centre [FGSC, http://www.fgsc.net/]. The strains were maintained and cultured on Vogel's medium (Vogel, 1956), i.e., Vogel's minimal salts supplemented with 1.5% sucrose and solidified with agar (Bacteriological agar, Biosolute, Germany). To collect conidia, cultures were grown on slants for two days at 30 °C in the dark, followed by five days under light/dark conditions to stimulate conidia formation.

For pathogenicity testing (in vivo experiments), we used *Aspergillus fumigatus* CCF6600 (Culture Collection of Fungi, Charles University) as a positive control. *A. fumigatus* conidia were obtained by culturing the fungus on Potato Dextrose Agar (PDA, Merck, Germany) slants for seven days at 37 °C.

### 2.2 Bioinformatic analysis

To determine the biological processes regulated by *CCG-8*, we performed Gene Ontology (GO) enrichment analysis on the list of *Neurospora crassa* genes regulated by *ccg-8* (NCU09686), as published in the supplementary data of (Hu et al., 2018). The analysis utilized the PANTHER database (https://pantherdb.org/) with the GO Biological Process Complete annotation dataset. Enrichment was determined using Fisher's exact Test with a False Discovery Rate (FDR) correction cutoff of <0.05.

### 2.3 Biomass preparation of *N. crassa*

For glycomic, lipidomic, and proteomic analyses, using sterile cotton swabs, we inoculated conidia of the wild-type (*N. crassa* wtA) and deletion mutant (*N. crassa* Δ*ccg-8*) strains onto Vogel plates covered with sterile cellophane. Vogel medium was solidified with agar in each experiment to mimic more natural conditions for the fungus (Víglaš and Olejníková, 2023). After 24 h of incubation at 30 °C in constant darkness (9:00–9:00 the next day), the fungal biomass was collected, lyophilized (snap-frozen by liquid nitrogen), and prepared for subsequent analysis.

To obtain large numbers of conidia, we followed the protocol described for the creation of deletion mutants of *N. crassa* (https://www.fgsc.net/neurosporaprotocols/KO_Protocols.pdf). The conidia of the tested strains were inoculated into 50 mL of Vogel agar (in 250 mL Erlenmeyer flask). We applied two flasks for *N. crassa* Δ*ccg-8*. Fungi were incubated for three days at 30 °C, followed by seven days at 25 °C in light-dark conditions. Conidia were then harvested using sterile distilled water, centrifuged (2500 ×*g* for 5 min), and the resulting pellet was lyophilized for use in further experiments.

### 2.4 Proteomic analysis of *N. crassa*

For proteomic analysis, proteins from dry biomass (mycelium or conidia of *N. crassa* in quadruplicates) were extracted into 100 μL of lysis buffer (2% SDS, 50 mM Tris-HCl, pH 7.8) for 15 min on a shaker at 1000 rpm and 90 °C. After centrifugation (10 min, 20,000 × *g*), the supernatant was used to determine protein concentration with the Pierce™ BCA Protein Assay Kit (Thermo Scientific, USA). For analysis, 30 μg of protein was reduced with 5 mM dithiothreitol for 30 min at 60 °C, followed by alkylation with 15 mM iodoacetamide for 20 min at 23 °C. Purification was performed using the SP3 protocol (Hughes et al., 2019), with proteins bound to Sera-Mag beads (GE Healthcare, USA) at a beads-to-protein ratio of 10:1. The purified proteins were resuspended in 50 μL of 100 mM tetraethylammonium bromide, pH 8.5, and digested with 0.5 μg of trypsin (1:60, *w*/*w*, Pierce™ Trypsin protease, MS-Grade, Thermo Scientific, USA) for 16 h at 37 °C. The eluted peptides were acidified with trifluoroacetic acid (TFA) to a final concentration of 1%, desalted using a microtip solid-phase extraction cartridge with C18 chromatographic silica resin (LiChroprep RP-18, Merck, Germany), and dried in a SpeedVac. Samples were resuspended in 20 μL of TA2 buffer (2% acetonitrile, 0.1% TFA), and the concentration of peptides was measured using a microvolume spectrophotometer.

One microgram of peptides was analyzed using a high-resolution Orbitrap Exploris 240 mass spectrometer (Thermo Fisher Scientific, USA) equipped with an EASY-Spray ion source, coupled to a UHPLC Vanquish Neo system (Thermo Fisher Scientific, USA). Peptides were desalted on a PepMap Neo trap C18 column (300 μm × 5 mm, 5-μm particle size; Thermo Scientific, USA) and separated using an EASY-Spray C18 analytical column, EASY-Spray PepMap Neo (75 μm × 500 mm, 2-μm particle size; Thermo Scientific, USA) with an integrated emitter. The two mobile phases used were: 0.1% formic acid in water (*v*/*v*) (A), and 80% acetonitrile (*v*/*v*) with 0.1% (*v*/*v*) formic acid in water (B). A linear gradient was applied at a flow rate of 250 nL/min with 2%–40% gradient of solution B for 120 min. Mass spectra were collected in data-dependent mode with a 2-s cycle at top speed. The parent scan ranging from 350 to 1700 *m*/*z* was recorded at a resolution of 120,000 (at *m*/*z* = 200). MS/MS scans were conducted with fragments acquired at a resolution of 30,000 and normalized collision energy of 30%.

Raw data files were processed using MaxQuant (version 2.7.0.0) (Cox et al., 2014) with the built-in Andromeda search engine. Search parameters included carbamidomethylation (C) as a fixed modification, and oxidation (M) and acetylation (protein N-terminus) as variable modifications. Trypsin was specified as the proteolytic enzyme, allowing a maximum of two missed cleavage sites and requiring a minimum peptide length of seven amino acids. The search was performed against the *Neurospora crassa* proteome FASTA [UniProt_ UP000001805_ Neurospora crassa (strain ATCC 24698 / 74-OR23-1 A / CBS 708.71 / DSM 1257 / FGSC 987)] downloaded on 24 October 2024. Statistical analysis of the identified and MaxLFQ quantified protein data was conducted using Perseus software (version 1.6.15.0). *t*-tests were performed using a permutation-based false discovery rate (FDR) of 0.005 and an S0 parameter of 0.1 to control for multiple comparisons and stabilize variance. For GO enrichment, *p*-values from individual Fisher's exact tests were adjusted using the Benjamini-Hochberg FDR procedure with False Discovery Rate (FDR) at a 0.02 threshold.

### 2.5 Lipidomic analysis of *N. crassa*

Total fatty acids profile and content was determined by method (Certik and Shimizu, 2000), with slight modifications. In brief, approximately 10–15 mg of lyophilized fungal biomass was accurately weighed and transferred into Pyrex glass tubes. Each sample was supplemented with 1 mL of the internal standard solution (C17:0, 0.1 mg/mL) in dichloromethane and 2 mL of methanolic HCl (2:1, *v*/v). The tubes were sealed and incubated at 50 °C for 3 h to allow transesterification. After incubation, samples were cooled to room temperature, followed by the addition of 1 mL of hexane and 1 mL of distilled water. The mixture was vortexed for 30 s and centrifuged at 5000 rpm for 5 min. The upper organic phase containing fatty acid methyl esters (FAMEs) was carefully collected and transferred to GC vials.

FAMEs were analyzed qualitatively and quantitatively by gas chromatography (Agilent 6890, USA) equipped with a flame ionization detector (FID) according to (Slaný et al., 2025).

Ergosterol was extracted from 0.2 g of dry mycelium by adding 3 mL of 60% KOH (*w*/*v*) in 50% methanolic solution and hydrolyzing the mixture at 70 °C for 2 h. Next, the sterols were extracted twice: first with 3 mL of n-hexane (shaking for 1 min), and then with 2 mL of n-hexane followed by centrifugation (5 min, 1300*g*). The combined organic phases were evaporated under a flow of nitrogen gas. The residue was dissolved in chloroform: methanol (2:1 *vol*.) and stored at −20 °C*. prior* to analysis, the solvent was evaporated and replaced with acetone. Measurements were performed on an Agilent 1100 High-Performance Liquid Chromatography (HPLC) instrument. Separation was achieved using an Eclipse XDB-C18 column (4.6 mm × 150 mm; particle size 5 μm) at 37 °C. The mobile phase was 95% methanol at a flow rate of 1.5 mL/min.

Two detectors were used: a Diode Array Detector (DAD) and a Corona Charged Aerosol Detector (CCAD). Ergosterol was identified by comparing its retention time and UV-VIS spectrum (DAD) to an ergosterol standard. Quantification was based on a calibration curve generated from known amounts of standard. The experiment was performed in triplicate and statistical significance was determined by an unpaired *t*-test.

### 2.6 Glycomic analysis of *N. crassa*

Approximately 0.1 g of lyophilized fungal biomass (mycelium) was processed according to the described protocols to obtain glucan and glycoprotein samples (Bzducha-Wróbel et al., 2024; Machová and Bystrický, 2012). In short, the *β*-glucan samples were isolated from freeze-dried biomass using extraction with 0.1 M NaOH solution at the first step. Samples were incubated in a water bath at 60 °C for 30 min. Then the extraction was repeated at 115 °C for 45 min. The next step was to rinse the sediment with distilled water while after, they were suspended in 0.1 M acetic acid. The samples were incubated at 85 °C water bath for 1 h. Glucan sediment was suspended in distilled water and washed 5 times. Lastly, the glucan suspension in water was freeze-dried. For glycoprotein isolation, the biomass of tested strains was subjected to extraction in an autoclave with 0.2 M NaCl at 120 °C for 1 h, three times. The combined supernatants were concentrated to approximately one-third of the initial volume and subsequently precipitated with three volumes of ethanol. The resulting precipitate was collected by sedimentation using a centrifuge, dissolved in distilled water, and dialyzed extensively against pure water. The dialyzed solution was finally lyophilized to yield the glycoprotein fraction. In the case of „whole cell“measurements, we used freeze-dried mycelium/conidia without any further processing (Adt et al., 2006; Dróżdż et al., 2025).

Elemental composition (C, H, N, S) of the isolated glucan samples was determined using a FLASH 2000 CHNS/O Organic Elemental Analyzer (Thermo Fisher Scientific, USA). The instrument was calibrated using acetanilide (Pragolab, Slovakia) as a standard. Instrument accuracy, verified with the standard, was within ±0.2%. Lyophilized samples were dried in desiccator over P_2_O_5_ prior to analysis. Approximately 1.5 mg of each sample was weighed for analysis. Measurements were performed in duplicates, and results are reported as mean ± SD and *t*-test was performed to clarify statistical significance.

FT-IR spectra were measured on NICOLET Magna 6700 (Thermo Fisher Scientific, USA) spectrometer with DTGS detector, experimental accessory – Smart Orbit and OMNIC 8.0 software. Infrared spectral analyses were carried out in mid-infrared region (from 4000 cm^−1^ to 400 cm^−1^) and spectral data obtained were presented as absorbance values. Solid-state measurements were performed using a Diamond Smart Orbit ATR accessory with 64 scans. Samples were measured in a biological duplicate. The resulting spectra aligned well, hence representative curves are presented in the results section.

ATR-FTIR spectra were processed using OMNIC 9.0. Spectral spacing was set to 2.0 cm^−1^, followed by spline baseline correction. Spectra were smoothed using a Savitzky–Golay filter (13 points) and vector-normalized. Second-derivative spectra were calculated using the Savitzky–Golay algorithm (13 points, third-order polynomial) and multiplied by −1 (inversed). Peak positions were identified using the Find Peaks tool. Fitting of spectral curves was done using OMNIC 9.0 in three regions separately: 3000–2800 cm^−1^; 1760–1480 cm^−1^; 1480–950 cm^−1^ [according to (Adt et al., 2006)]. The accuracy of the fit is given by the *F*-statistics value. Final plots were generated in Microsoft Excel. All fittings yielded *F*-values below the critical threshold, indicating high accuracy, with the exception of the mycelial Δ*ccg-8* whole-cell measurement in the 1480–950 cm^−1^ region *F* = 4.98, *p* = 0.026). However, the effect size associated with the lack-of-fit was negligible (η^2^ ≈ 0.016), indicating that the residual error (the discrepancy between the measured spectrum and the modeled spectrum) accounts for only ≈1.6% of the total spectral variance. This confirms that the overall goodness-of-fit remained robust, and the minor residual error did not significantly affect the integrated peak areas used for subsequent comparative analysis.

### 2.7 Susceptibility testing

To assess the in vitro role of deletion of gene *ccg-8*, we performed cultivation tests to determine the growth profile of *N. crassa* wtA and *N. crassa* Δ*ccg-8*. We exposed the strains to various stress agents: (*i*) NaCl [Mikrochem, Slovakia], Sorbitol [Sigma Aldrich, USA] – causing osmotic stress; (*ii*) Tween-80 [Biolife, Italy] – causing plasma membrane stress; (*iii*) Congo Red [Erba Lachema, Czech Republic] – causing cell wall stress; (*iv*) tunicamycin [Sigma Aldrich, USA] – endoplasmic reticulum stress; and (*v*) antifungals – azoles: Propiconazole, Posaconazole, Voriconazole, Ketoconazole (Sigma Aldrich, USA); and echinocandins: Caspofungin, and Micafungin (Sigma Aldrich, USA) – though they may be also considered as plasma membrane and cell wall stress reagents, respectively.

The susceptibility tests followed a previously published protocol (Víglaš and Olejníková, 2023). Osmotic and Cell Surface stress agents (*i-iii*) were added to the Vogel medium before autoclaving and aseptically poured into 5 cm Petri dishes (5 mL total). Tunicamycin and solutions of antifungals (prepared in DMSO) were aseptically pipetted to a 5 cm-diameter Petri dish (50 μL) and dissolved in 5 mL of autoclaved culture medium (resulting in 1% *v*/*v* DMSO). Control plates contained reagent-free medium (for osmotic/cell surface agents) or 1% *v*/*v* DMSO (for tunicamycin/antifungals). Plates were inoculated and incubated at 30 °C in the dark until the control colony reached the edge of the plate. Then the percentage (%) of growth was calculated: diameter of colony in the presence of reagent /diameter of control colony × 100%. We plotted detected results and observed trends in susceptibility profiles. Each test was repeated at least three times with inoculation performed in the same time of the day.

### 2.8 Larval culture and inoculation

Greater wax moth larvae (*Galleria mellonella*) were reared in our laboratory settings at 30 °C until they reached the final instar stage, weighing approximately 0.2–0.3 g. Larvae were fed an artificial diet three times per week (Segéňová et al., 2025). Once prepared, they were randomly allocated into experimental groups of ten individuals each.

Fungal conidia were prepared based on the procedure described in Section 2.3, with one modification: conidia were harvested using Phosphate Buffered Saline (PBS, pH 7.2) supplemented with 0.01% Tween-80. The final conidia pellet was resuspended in 5 mL of PBS + Tween-80 to obtain the final concentration 1 × 10^7^ conidia per mL (dose of 1 × 10^5^ per larva) for *N. crassa* wtA and *N. crassa* Δ*ccg-8.* The positive control for pathogenicity, *A. fumigatus* CCF6600, was harvested directly from PDA slants and adjusted to a maximum concentration of 5 × 10^7^ conidia per mL (dose 5 × 10^5^ conidia per larva).

Inoculation of larvae was performed by injecting 10 μL of the respective conidial suspension in PBS containing Tween-80 into the last proleg of each larva. Three negative control groups were included: (*1*) uninoculated larvae to assess background mortality, (*2*) larvae injected with PBS to account for the effect of physical trauma, and (*3*) larvae injected with PBS-Tween-80 to evaluate any potential impact of Tween-80. Following inoculation, larvae were incubated at 30 °C and monitored daily for survival over seven days. Mortality was defined as the absence of movement in response to gentle stimulation with tweezers. The experiment was conducted in four biological replicates.

To verify fungal viability prior to larval injection, conidial suspensions were processed as follows: first, conidial density was determined using a Bürker counting chamber; second, the concentration was adjusted to 100 conidia/mL; finally, 250 μL of the suspension was plated (pipetted and spread) onto selective media. *N. crassa* (wild type and deletion mutant) was cultured on FGS agar prepared according to standard *Neurospora* KO protocols (https://www.fgsc.net/neurosporaprotocols/KO_Protocols.pdf), while *A. fumigatus* was plated on Sabouraud Dextrose Agar supplemented with chloramphenicol (Merck, Germany) and 0.01% (volume/volume) Triton X-100 (Sigma-Aldrich, USA). The inclusion of sorbose (in FGS agar) and Triton X-100 served to restrict colony expansion, facilitating accurate enumeration of small, discrete colonies.

To assess the fungal state within the larvae 48 h post-injection, the posterior segments of the larvae were excised, and internal fluids and tissues were pressed (by squeezing the larvae) onto Potato Dextrose Agar (PDA, Merck, Germany) plates. These plates were incubated at 30 °C for 48 h and monitored visually for fungal growth.

### 2.9 Fluorescent imaging of nodules containing conidia

To assess the fate of conidia (*N. crassa* wtA, *N. crassa* Δ*ccg-8* and *A. fumigatus* CCF6600 for positive control), we extracted the structures called „nodules“, where conidia are encapsulated in larval body. Specifically, we froze larvae 3-, 24-, and 48-h post-injection by brief immersion in liquid nitrogen and then fixed in 4% formaldehyde (pH 6.9 in PBS) for 24 h. The subsequent procedure followed (Sheehan et al., 2018). Larvae were dissected, and the nodules were carefully transferred onto a glass slide using fine needles. Nodules were then crushed and stained with Calcofluor White for 15 min at room temperature. The excess stain was removed by suction using filter paper, and the slide was washed with 0.9% NaCl solution. Nodules were immediately examined using a microscope Axio Imager A1 (Zeiss, Germany) – 400 × magnification, fluorescence detected using excitation/emission wavelength 365/440 nm (X-Cite 120Q lamp, Excelitas Technologies, USA) with photo documentation equipment Axiocam ICC 1 and AxioVision 4.8 software. The experiment was repeated three times. The nodules were extracted from three larvae at each time point.

## 3 Results

### 3.1 Transcription factor CCG-8 is important for the cell surface landscape of *N. crassa*

To decipher the impact of Δ*ccg-8*, we performed an in silico analysis of a published dataset of the transcriptional regulatory network containing 1011 genes regulated by the *CCG-8* transcription factor (Hu et al., 2018). Results are presented in Table 1 and Supplementary Table S1. Gene enrichment analysis of the dataset suggests a role for *CCG-8* in the formation of cell surface structures. The broader term GO:0071852 (fungal-type cell wall organization or biogenesis) was selected for presentation in Table 1 because it encompasses the greatest number of cell wall-related genes regulated by the product of *ccg-8*. This finding indicates that the Δ*ccg-8* deletion likely affects *N. crassa* at the cell surface level.

**Table 1:** GO enrichment results (biological process) for a set of genes indicated as regulated by transcription factor *CCG-8* [full results, Table S1, gene dataset adopted from (Hu et al., 2018)].

GO ID	GO Term	Fold enrichment	Adjusted *p*-value
GO:0070592	cell wall polysaccharide biosynthetic process	4.94	4.11E−02
GO:0071852	fungal-type cell wall organization or biogenesis	3.13	9.40E−04
GO:0006031	chitin biosynthetic process	4.94	4.04E−02
GO:1903338	regulation of cell wall organization or biogenesis	4.94	4.27E−02
GO:0006629	lipid metabolic process	2.22	1.06E−08
GO:0008610	lipid biosynthetic process	2.27	6.10E−05
GO:0008654	phospholipid biosynthetic process	2.21	3.54E−02
GO:0006396	RNA processing	0.54	3.67E−02
GO:0006259	DNA metabolic process	0.28	1.43E−03

### 3.2 Deletion Δccg-8 impacts *N. crassa* at a wide proteomic scale

Proteomic analysis confirmed the importance of the transcription factor encoded by gene *ccg-8*, revealing significant differences between the wild-type strain and deletion mutant. We detected over 1400 dysregulated proteins in the mycelium and 1107 dysregulated proteins in conidia samples. Of these, 414 proteins showed altered expression in both sample types. The full proteomic data are available in the PRIDE repository and Supplementary Table S2.

**Fig. 1:**
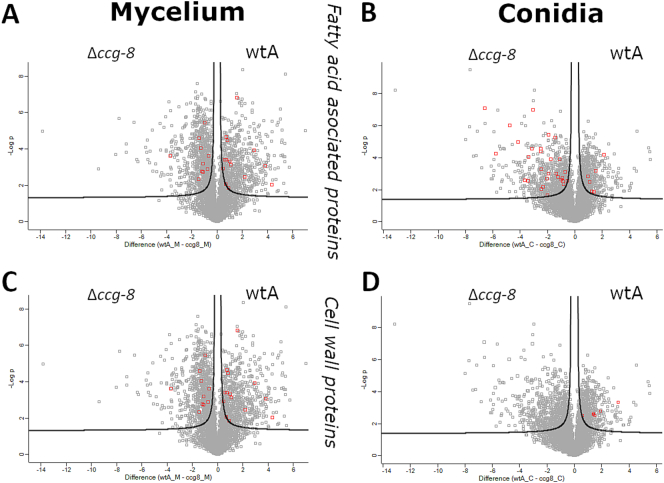
Volcano plots comparing the proteomic profiles of *N. crassa* wtA and *N. crassa Δccg-8* strains. The log*2* fold change (difference) is plotted on the X-axis, and the statistical significance *(−log10*, i.e., adjusted *p*-value) is plotted on the Y-axis. Statistically significant proteins (FDR < 0.005) are located in the upper-left and upper-right quadrants, separated from non-significant signals by black hyperbolic curves. [A], [B] – Red points highlight proteins associated with “fatty acid” biological processes (GO terms). [C], [D] – Highlighted points represent differentially expressed cell wall proteins, with localization based on (Patel and Free, 2019). [A] and [C] represent mycelia samples; [B] and [D] represent conidia samples. Positive values indicate higher abundance *N. crassa* wtA, negative value means upregulation in *N. crassa* Δ*ccg-8. (For interpretation of the references to colour in this figure legend, the reader is referred to the web version of this article.)*

To characterize the functional roles of these proteins in biological processes, we performed enrichment analysis (Table 2, Table S2). The detected dysregulated proteins are involved in diverse biological processes, ranging from rRNA processing and ribosome biogenesis to fatty acid metabolism (enriched in conidia, but proteins handling fatty acids are present in the mycelia sample as well; Fig. 1A, B). While not formally enriched as a category, several proteins critical to cell wall biosynthesis and structural integrity were nonetheless dysregulated, particularly when comparing the *N. crassa* wild-type mycelium to the Δ*ccg-8* mutant (Fig. 1C, D).

**Table 2:** GO enrichment results (biological process) for a detected set of dysregulated proteins between *N. crassa* wtA and *N. crassa* Δ*ccg-8* (for full results, see Table S2).

Mycelium sample			
GO ID	GO Term	Fold enrichment	Adjusted *p*-value
GO:0000466	maturation of 5.8S rRNA from tricistronic rRNA transcript (SSU-rRNA, 5.8S rRNA, LSU-rRNA)	4.95	3.13E−04
GO:0000463	maturation of LSU-rRNA from tricistronic rRNA transcript (SSU-rRNA, 5.8S rRNA, LSU-rRNA)	4.71	3.10E−10
GO:0042273	ribosomal large subunit biogenesis	3.90	4.74E−05
GO:0000447	endonucleolytic cleavage in ITS1 to separate SSU-rRNA from 5.8S rRNA and LSU-rRNA from tricistronic rRNA transcript (SSU-rRNA, 5.8S rRNA, LSU-rRNA)	3.64	3.26E−04
GO:0000472	endonucleolytic cleavage to generate mature 5′-end of SSU-rRNA from (SSU-rRNA, 5.8S rRNA, LSU-rRNA)	3.63	4.67E−03
GO:0000462	maturation of SSU-rRNA from tricistronic rRNA transcript (SSU-rRNA, 5.8S rRNA, LSU-rRNA)	3.16	8.21E−04
GO:0000480	endonucleolytic cleavage in 5’-ETS of tricistronic rRNA transcript (SSU-rRNA, 5.8S rRNA, LSU-rRNA)	3.54	1.54E−02
GO:0006364	rRNA processing	3.43	5.86E−11
GO:0006412	translation	2.60	7.14E−10
*Conidia sample*			
GO ID	GO Term	Fold enrichment	Adjusted *p*-value
GO:0006096	glycolytic process	7.56	1.46E−04
GO:0006635	fatty acid beta-oxidation	5.02	1.46E−04

### 3.3 Altered lipid composition in the Δccg-8 mutant indicates disrupted membrane homeostasis

**Fig. 2:**
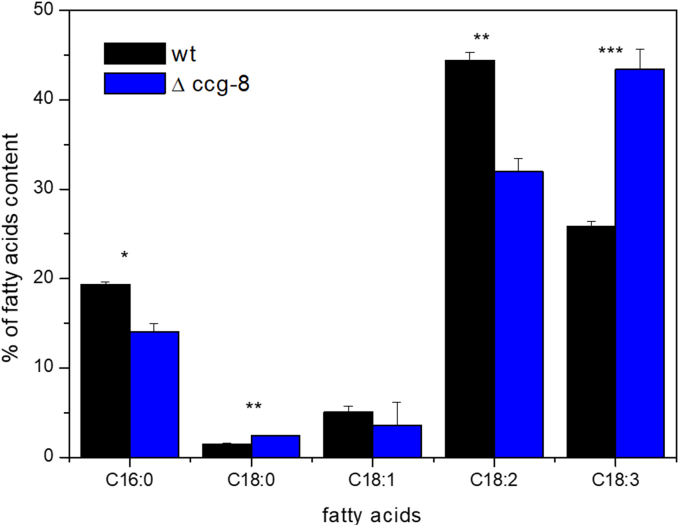
Relative abundance of major fatty acids in the mycelium of *Neurospora crassa* wild-type (WT) and Δ*ccg-8* strains. The figure shows the mean ± standard deviation (SD) of five predominant fatty acids: palmitic acid (C16:0), stearic acid (C18:0), oleic acid (C18:1Δ9), linoleic acid (C18:2Δ9,12), and linolenic acid (C18:3Δ9,12,15). Measurement comes from three independent biological replicates. Statistical significance of differences between WT and Δ*ccg-8* was determined using the Student's test (*p < 0.05; **p < 0.01; ***p < 0.001).

Comparative analysis of major fatty acid profiles in the mycelial biomass revealed significant differences between the wild-type strain and the Δ*ccg-8* mutant (Fig. 2). The mutant strain showed a pronounced increase in α-linolenic acid (C18:3Δ9,12,15), reaching 40.97 ± 4.07% (of all fatty acids) compared to 25.82 ± 0.51% in the wild type. Conversely, the relative abundance of linoleic acid (C18:2Δ9,12) was significantly reduced in Δ*ccg-8* mutant (35.20 ± 5.43%) versus the wild type (44.42 ± 0.83%). Levels of oleic acid (C18:1Δ9) also declined (2.80 ± 2.11% in Δ*ccg-8* vs. 5.03 ± 0.71% in WT).

Minor shifts were observed in saturated fatty acids: stearic acid (C18:0) slightly increased, while palmitic acid (C16:0) was reduced in Δ*ccg-8*, albeit with higher inter-replicate variability.

These alterations reflect a major shift in fatty acid desaturation and elongation pathways in the absence of *CCG-8*, causing an imbalance among saturated, monosaturated, and polyunsaturated fatty acids. The substantial increase in the polyunsaturated fatty acid C18:3, alongside the concurrent reduction in C18:2 and C18:1, strongly suggests deregulated desaturase activity and altered membrane lipid composition. This conclusion is further supported by ergosterol analysis, which showed that the mycelium of Δ*ccg-8* deletion mutant contains significantly less ergosterol than the wild-type strain (Table 3).

**Table 3:** The ergosterol content of the mycelium of *N. crassa.*

ng of sterol / g of dry mycelium	
*N. crassa* wtA	*N. crassa* Δ*ccg-8*
15.346 ± 3.384	8.600 ± 0.618

The two-tailed *P* value equals 0.0502.

### 3.4 Changed glycomic profile in deletion mutant as the main determinant of “antigenic structures” at the cell surface

Next, we carried out a basic glycomic analysis of *N. crassa* biomass to further investigate the impact of *ccg-8* deletion. Elemental analysis of the isolated glycoprotein and glucan fractions showed no significant differences between the wild type and the Δ*ccg-8* mutant (Table 4).

**Table 4:** Yield (*Y*) and elemental composition analysis of the isolated glycoprotein and glucan samples of *N. crassa* wtA and *N. crassa* Δ*ccg-8*, respectively (no statistical difference detected in composition). Values are given as mean ± SD.

Sample	*Y* [%]	N [%]	C [%]	H [%]	S [%]
wtA glycoprotein	0.50 ± 0.21	6.5 ± 1.0	37.6 ± 1.1	6.1 ± 0.2	0.6 ± 0.2
Δ*ccg-8* glycoprotein	0.26 ± 0.09	7.7 ± 0.9	37.4 ± 1.0	6.0 ± 0.2	1.1 ± 0.2
wtA glucan	1.20 ± 0.36	0.9 ± 0.2	41.2 ± 0.4	6.5 ± 0.0	0.0 ± 0.0
Δ*ccg-8* glucan	0.78 ± 0.06	0.8 ± 0.0	41.4 ± 0.3	6.7 ± 0.3	0.0 ± 0.0

**Fig. 3:**
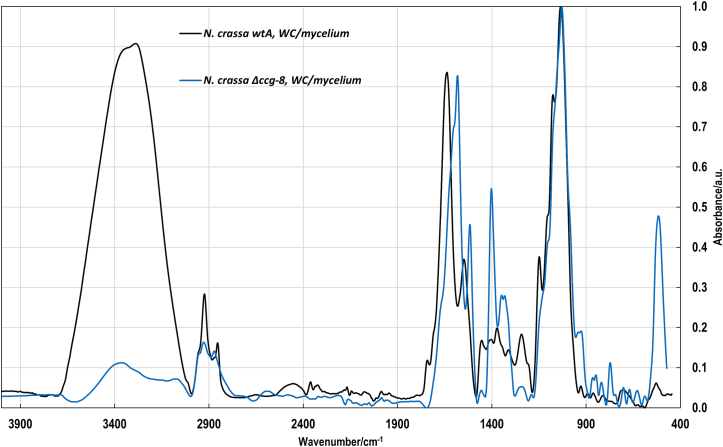
ATR-FTIR spectra of whole-cell (WC) mycelium samples. Comparative spectra of lyophilized *N. crassa* wtA (black line) and *N. crassa* Δ*ccg-8* (blue line) are shown across the full mid-infrared range. Significant biochemical alterations are observed in the mutant, particularly within the protein/amide region (1700–1500 cm^−1^) and the polysaccharide fingerprint region (1200–900 cm^−1^). (For interpretation of the references to colour in this figure legend, the reader is referred to the web version of this article.)

ATR-FTIR spectra of dry whole cells, isolated glycoprotein, and glucan identified characteristic absorption bands corresponding to key biochemical components (lipids, chitin, *α*-glucans, *β*-glucans, and glycoproteins) (Table S4). The presence and intensity of specific bands (e.g., in the fingerprint and skeletal regions) highlight structural and compositional variations that may reflect genetic or phenotypic changes relevant for fungal biology (Fig. 3).

For better understanding changes, we compared data obtained from fitting of the selected spectral regions (Fig. S6) belonging to lipids, proteins and carbohydrates according to published method (Adt et al., 2006). The obtained band areas were normalized to have sum of 100 in whole inspected area. Results are summarised in Table S4. The listed peaks were assigned according to referenced literature (Adt et al., 2006; Bikmurzin et al., 2022; Dróżdż et al., 2025; Hsu et al., 2010; Novák et al., 2012). Next, we calculated: *v*_as_(CH_2_)/*v*_as_(CH_3_) and v_s_(CH_2_)/v_s_(CH_3_) ratios [*v*_as_ and *v*_s_ mean asymmetric/symmetric stretching vibrations of C—H bonds in the fatty acid tails of lipids] to provide changes in lipid chain length, branching and/or saturation level; Amide II/Amide I ratio to provide changes in protein structure; *β*-sheet/*α*-helix ratio to compare secondary structure of proteins; protein-to-glycan ratio (Table 5) and Pearson correlation coefficient to compare pairs of fitted peak areas (Table S4).

**Table 5:** Quantitative analysis of curve-fitted major component bands of the FTIR spectra of *N. crassa* from “whole cell” measurement.

Peak area ratio	Mycelium		Conidia		Possible changes
	*N. crassa* wtA	*N. crassa* Δ*ccg-8*	*N. crassa* wtA	*N. crassa* Δ*ccg-8*	
*v*_as_(CH_2_)/*v*_as_(CH_3_)	5.09	5.61	2.95	3.48	lipid chain length, branching and/or saturation level
*v*_s_(CH_2_)/*v*_s_(CH_3_)	1.03	0.54	0.50	0.53	
Amide II/Amide I	0.32	0.65	0.53	0.52	protein structure
*β*-sheet/*α*-helix	1.72	NA	2.70	NA	secondary structure of proteins
protein/glycan	0.68	0.89	0.80	0.80	Proteins, polysaccharides

NA = not applicable; The denominator (peak area) was not detectable (is zero) in sample, preventing ratio calculation.

Conidial spectra exhibited only minor differences between *N. crassa* wtA and *N. crassa Δccg-8* (Table 5). Notably, the protein-to-glycan ratios were identical (0.80 for both strains), and the Amide II/Amide I ratios were nearly unchanged (0.53 and 0.52 for wild-type and deletion mutant, respectively). In contrast, mycelial spectra showed pronounced differences. The protein-to-glycan ratios were 0.68 for *N. crassa* wtA and 0.89 for *N. crassa Δccg-8*, while the Amide II/Amide I ratios were 0.32 and 0.56, respectively. The altered band pattern is further supported by the Pearson correlation coefficient derived from the fitted spectra: *r* ≈ 0.16 for mycelial cell walls, indicating substantial spectral divergence, compared with *r* ≈ 0.98 for conidia, where spectra were highly similar. Variations, in the Amide I/II and *β*-sheet/*α*-helix regions, may indicate possible modifications in cell surface molecular composition and/or structural organization of surface-associated proteins.

### 3.5 The deletion of ccg-8 increases the susceptibility of *N. crassa* to various stress agents

To assess the phenotypic impact of the detected molecular differences between the *N. crassa* wild-type (wtA) and Δ*ccg-8* mutant strains, we performed susceptibility studies using agents that target different cellular structures.

**Fig. 4:**
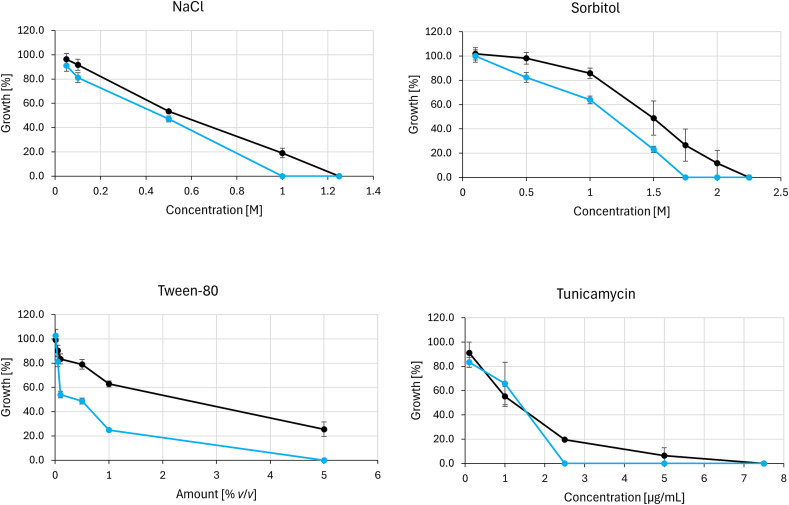
The susceptibility of *N. crassa* wild-type A and *N. crassa* Δ*ccg-8* to agents causing osmotic stress (NaCl, Sorbitol), plasma membrane stress (Tween-80), and endoplasmic reticulum stress (Tunicamycin) measured as the percentage of growth in the presence of various concentrations of stress-inducing agents. Deletion mutant *N. crassa* Δ*ccg-8* displayed elevated susceptibility throughout the panel. **Black** line = *N. crassa* wtA,  line = *N. crassa* Δ*ccg-8. (For interpretation of the references to colour in this figure legend, the reader is referred to the web version of this article.)*

For the first panel of compounds, we used (*i*) osmotically active NaCl and sorbitol, affecting cell wall and plasma membrane, (*ii*) Tween-80, which mainly impacts plasma membrane integrity, though GPI-anchored cell wall proteins are affected naturally as well, and (*iii*) tunicamycin, triggering endoplasmic reticulum stress by inhibition of *N*-glycosylation of proteins (Fig. 4). The Δ*ccg-8* mutant was more susceptible to all tested compounds, with the difference becoming most pronounced at higher concentrations. The wild-type strain retained slight residual growth where the deletion mutant showed complete growth inhibition (Fig. S1).

**Fig. 5:**
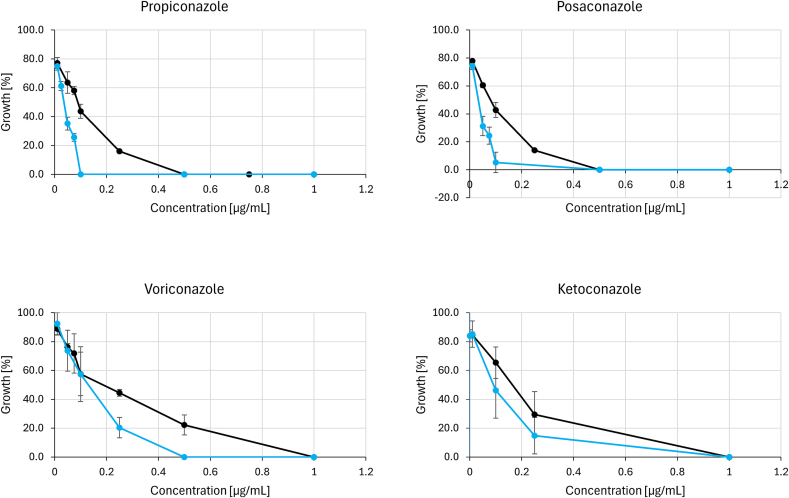
The susceptibility of *N. crassa* wild-type A and *N. crassa* Δ*ccg-8* to azole antifungals measured as the percentage of growth in the presence of various concentrations of azoles. Deletion mutant *N. crassa* Δ*ccg-8* is more strongly inhibited in the presence of propiconazole and posaconazole, while slightly increased susceptibility is also visible in the presence of ketoconazole. **Black** line = *N. crassa* wtA,  line = *N. crassa* Δ*ccg-8. (For interpretation of the references to colour in this figure legend, the reader is referred to the web version of this article.)*

The second panel comprised azole antifungals (propiconazole, ketoconazole, posaconazole, and voriconazole) that inhibit ergosterol biosynthesis (Fig. 5). Deletion of *ccg-8* increased susceptibility to all azoles, particularly posaconazole. The heightened susceptibility to ketoconazole is consistent with previous publications (Sun et al., 2014; Xue et al., 2019). In the instance of voriconazole, the concentration of 0.5 μg/mL resulted in complete growth inhibition for Δ*ccg-8*, while the wild type retained residual growth.

**Fig. 6:**
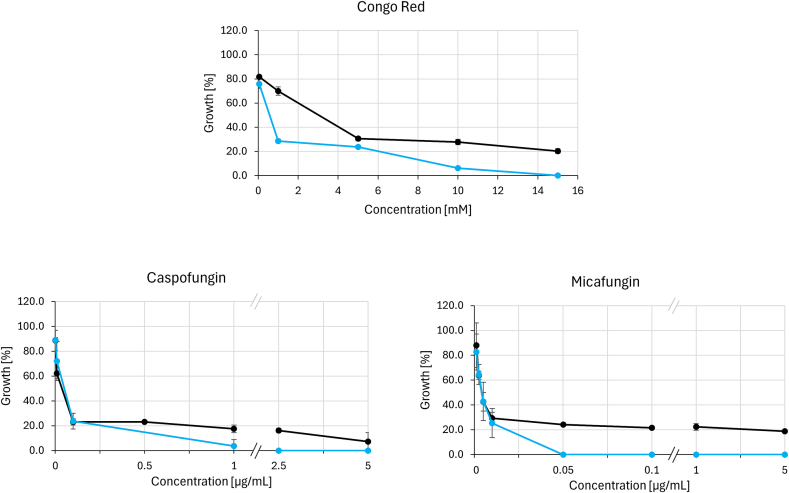
The susceptibility of *N. crassa* wild-type A and *N. crassa* Δ*ccg-8* to cell wall stress agents (Congo red and echinocandin antifungals) measured as the percentage of growth in the presence of various concentrations of these agents. Deletion mutant *N. crassa* Δ*ccg-8* seem to be less capable of tolerating higher concentrations of tested compounds. **Black** line = *N. crassa* wtA,  line = *N. crassa* Δ*ccg-8. (For interpretation of the references to colour in this figure legend, the reader is referred to the web version of this article.)*

The final panel consisted of cell wall stress agents (Fig. 6): Congo red (binds to chitin) and echinocandin antifungals (inhibit *β*-glucan biosynthesis). The *N. crassa* wild-type strain proved highly tolerant to echinocandins, maintaining a residual growth of roughly 20% across the tested concentration range. The deletion of *ccg-8* abolished this tolerance phenotype, further supporting a role of the transcription factor in general adaptation to stress conditions.

### 3.6 The deletion of the transcription factor encoded by gene ccg-8 affects the clearance of *N. crassa* from *Galleria mellonella* larvae

It is generally accepted that xenobiotic particles stimulate the innate immune system in living organisms. We hypothesized that if the surface structures of *N. crassa* are modified by the Δ*ccg-8* deletion, the innate immunity of *G. mellonella* larvae should react differently, even though *N. crassa* is a non-pathogen.

**Fig. 7:**
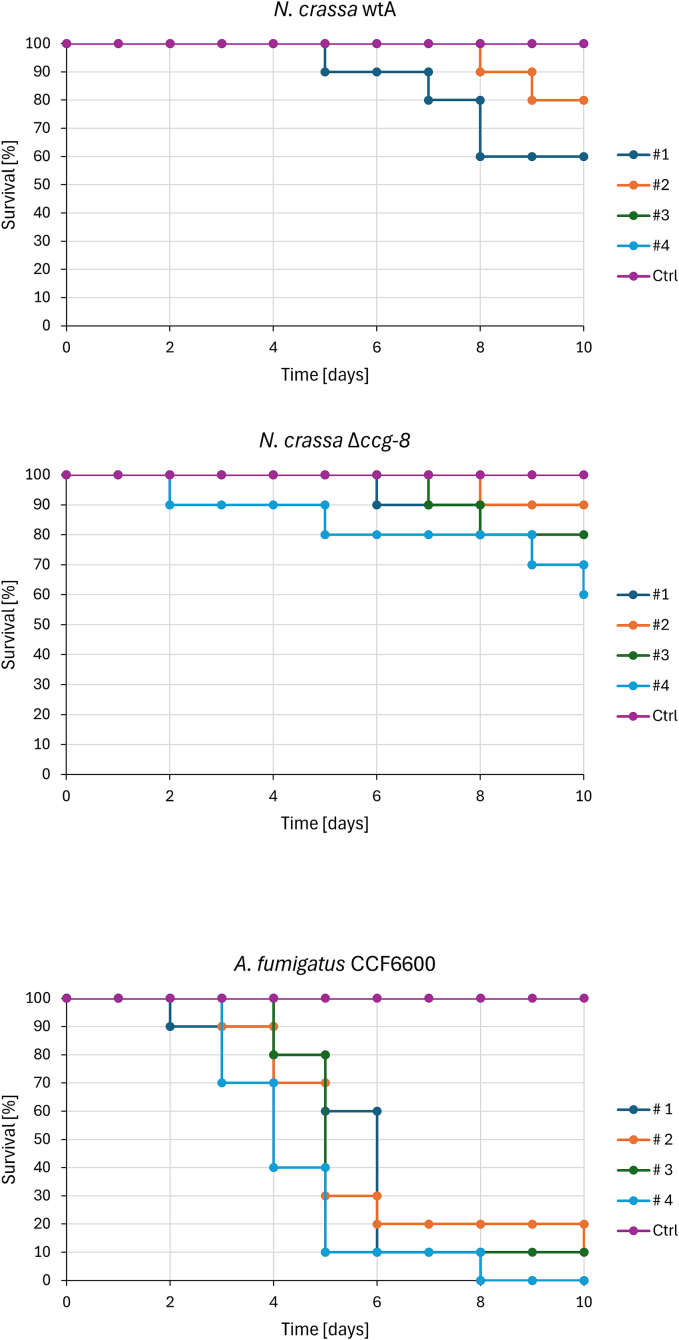
The survival of *G. mellonella* larvae after being injected with conidia of *N. crassa* wtA, *N. crassa* Δ*ccg-8*, and *A. fumigatus* CCF6600. Applied dose: 1 × 10^5^ conidia per larva for *N. crassa*, 5 × 10^5^ conidia per larva for *A. fumigatus*. Data from four independent trials are shown [#1 - #4]. Larvae in the control group were injected with 10 μL PBS with 0.01% Tween-80. Note that the survival curves for *A. fumigatus* are highly consistent between replicates, whereas the *N. crassa* curves show greater dispersion.

To test this, first, we injected conidia of the *N. crassa* wtA and Δ*ccg-8* strains into *G. mellonella* larvae, using the pathogenic *A. fumigatus* CCF6600 as a positive control (Fig. 7). The viability of conidia was checked prior to injection (Fig. S4). While *A. fumigatus* consistently caused larval mortality over time (Fig. 7), the survival rates for Δ*ccg-8* and wtA strains varied, showing no clear distinction between the two *N. crassa* strains. However, larvae injected with Δ*ccg-8* conidia melanized more rapidly than those treated with wtA (Fig. S2). *A. fumigatus*-infected larvae showed melanization comparable to the control. This suggested that earlier immune changes occur upon exposure to the Δ*ccg-8* mutant.

To clarify the fate of conidia, we dissected the larvae and isolated the nodules – particles where conidia are encapsulated by immune cells (oenocytoids), which trigger melanization and ROS production to eliminate the foreign particle (Pereira et al., 2018). After dissection, the nodules presumably containing conidia of *N. crassa* were of very fine structure, floccule-like (Fig. S3). They were more abundantly present in the case of larvae applied with *N. crassa* Δ*ccg-8* (probably responsible for higher melanization compared to *N. crassa* wtA). In contrast, nodules of *A. fumigatus* were more spherical and sparser.

**Fig. 8:**
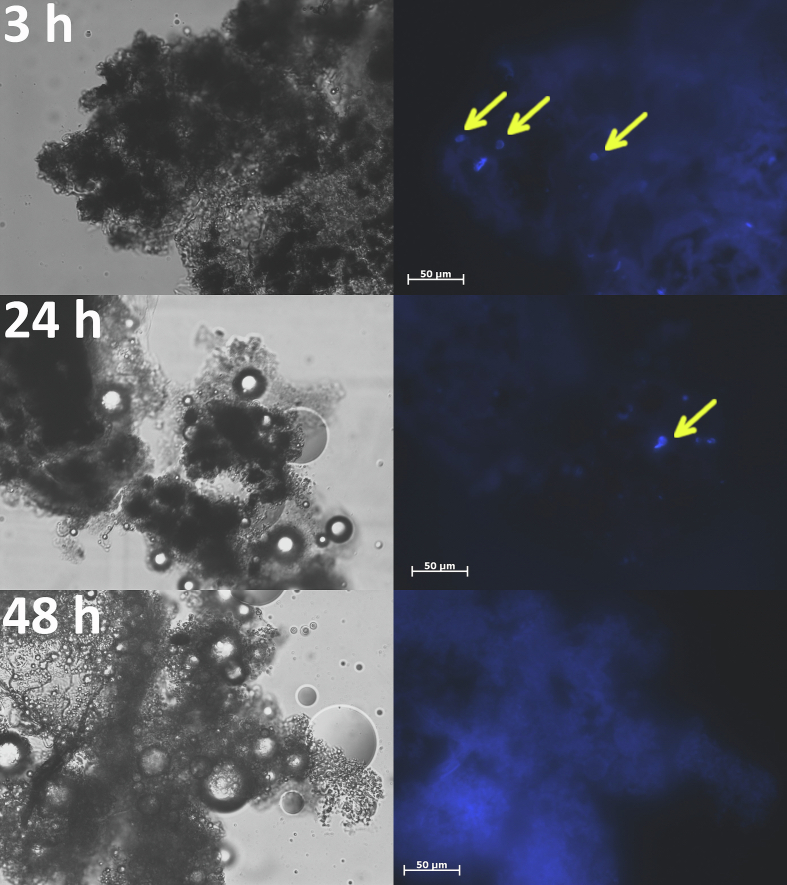
Clearance of conidia of *N. crassa* wtA from the nodules of *Galleria mellonella* larvae, 3 h, 24 h, and 48 h post-injection. Conidia (indicated by arrow) were present in small numbers in nodules extracted 24 h post-injection. Calcofluor white stained nodules were visualized by 400× magnification (excitation/emission 350/461 nm).

**Fig. 9:**
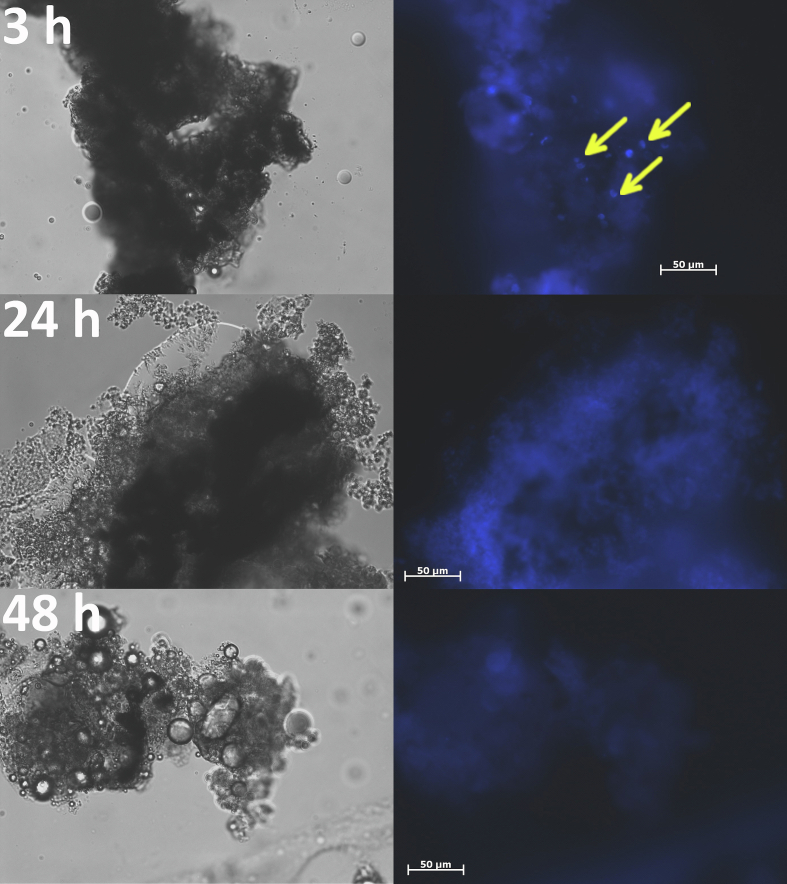
Clearance of conidia of *N. crassa* Δ*ccg-8* from the nodules of *Galleria mellonella* larvae, 3 h, 24 h, and 48 h post-injection. Conidia (indicated by arrow) were last present in nodules extracted 3 h post-injection. Calcofluor white stained nodules were visualized by 400× magnification (excitation/emission 350/461 nm).

**Fig. 10:**
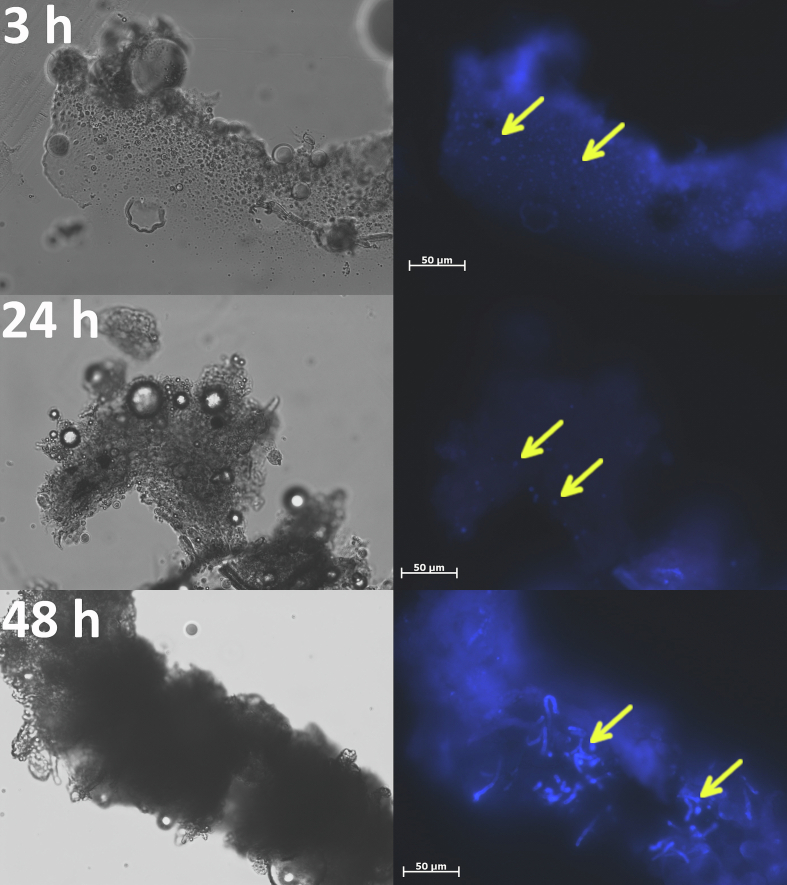
The infection process by conidia of *A. fumigatus* CCF6600 in the *Galleria mellonella* larvae, 3 h, 24 h, and 48 h post-injection. Conidia (indicated by arrow) survived the effect of the innate immune system of larvae in nodules, and after 48 h post-infection, they germinated. Calcofluor white stained nodules were visualized by 400× magnification (excitation/emission 350/461 nm).

Calcofluor white staining of nodules (Fig. 8, Fig. 9) confirmed that the conidia of *N. crassa* were cleared out of the larval body. The conidia of *N. crassa* Δ*ccg-8* appeared to be eliminated more rapidly, present at 3 h post-injection but undetectable by 24 h. A few conidia of *N. crassa* wtA remained at 24 h, though empty nodules predominated. Conversely, conidia of *A. fumigatus* tolerated the stress conditions inside nodules and germinated by 48 h (Fig. 10), which likely caused the subsequent drop in larval survival after 3 days (Fig. 7); the hyphae breached the nodules and damaged tissue. Finally, we confirmed the clearance by plating larval tissues/fluids onto fresh medium; no growth of *N. crassa* was detected after 48 h, while *A. fumigatus* was growing (Fig. S5).

These findings collectively demonstrate that *N. crassa* is effectively removed by the larval immune system and prove that Δ*ccg-8* deletion leads to an accelerated immune recognition (faster melanization and clearance). Furthermore, this establishes the *G. mellonella* model as a new avenue for in vivo research of *N. crassa* and other non-pathogenic filamentous fungi.

## 4 Discussion

*Neurospora crassa* is an essential and well-established model organism for studying filamentous fungi. Its extensive history of research spans basic mycology (Patel and Free, 2019; Weichert et al., 2020), biotechnological uses (Havlik et al., 2017), and, more recently, investigations into response to antifungals (Zhou et al., 2022). A significant research focus has been on elucidating the roles of its many transcription factors in response to the azole drug, ketoconazole (X. Chen et al., 2016; Xue et al., 2019; Yin et al., 2021). While these published works give us a glimpse into stress adaptation in this non-pathogenic organism, a broader contextualization of these findings is often lacking. Our work aimed to expand this knowledge by focusing on the *N. crassa* transcription factor encoded by the *ccg-8* gene. Specifically, we aimed to determine why its deletion increases susceptibility to ketoconazole and to evaluate the feasibility of analyzing this non-pathogenic fungus using the in vivo model *Galleria mellonella*, a common host for pathogenic microorganisms.

The literature previously hinted at the broader role of *CCG-8* (Hu et al., 2018). The dataset of genes regulated by this transcription factor shows abundant targets with Gene Ontology terms pointing to fungal cell wall structure and lipid/phospholipid metabolism (Table 1, Table S1). This suggested that the Δ*ccg-8* deletion would significantly affect the cell surface structures of the filamentous fungus.

However, our proteomic analysis yielded a different pattern (Table 2). We did not detect an enrichment of cell wall-related proteins. Nonetheless, individual members known to be cell wall constituents were still identified among the dysregulated proteins. These proteins are highlighted in Fig. 1 (Table S3). The most pronounced finding in the proteomic dataset was the enrichment of dysregulated proteins responsible for ribosome biogenesis (Table 2). This difference may arise because transcript levels do not always correlate with protein abundance due to post-transcriptional modifications, translation efficiency, or protein turnover rates. Therefore, if ribosome assembly was still underway at the point of analysis, the final downstream targets of *CCG-8*, such as cell wall proteins, may not have been affected to a scale detectable by enrichment analysis. Moreover, the transcriptional dataset reveals that the targets for *CCG-8* were identified exclusively through TF-perturbation (RNA-seq) rather than direct binding assays (e.g., ChIP-seq). Consequently, the reported regulon likely captures a significant number of indirect downstream effectors rather than solely direct targets, which further explains the divergence between the transcriptomic predictions and our proteomic landscape (where the detection of indirect target is also possible).

The observed impact on ribosome formation is structurally consistent with the Gene Ontology (GO) annotation of the *ccg-8* gene, which is linked to the translation processes and the Endoplasmic Reticulum Unfolded Protein Response (UPR) [https://www.ebi.ac.uk/QuickGO/annotations?geneProductId=Q01306]. Regarding the former, *ccg-8* is a well-known clock-controlled gene (Dong et al., 2008). For a filamentous fungus growing on a solid surface, the daily cycle presents a predictable series of stressors, such as oxidative or osmotic stress. Consequently, the fungus rhythmically channels metabolic energy into defense systems during periods of peak environmental stress (e.g., synchronization with sunrise, which increases the risk of desiccation). This rhythmic governance extends to numerous mechanisms responsible for stress adaptation (Coca-Ruiz and Boy-Ruiz, 2025), including ribosome biogenesis (Ding et al., 2021). Accounting for this rhythmic variation, we performed the inoculation and harvesting of fungal biomass to the same time for both, wild-type and Δ*ccg-8* mutant. Our detection of dysregulated proteins related to ribosome biogenesis (Table 2, Table S2) suggests a disruption in these rhythmic processes, also presumably within the machinery required for synthesizing stress-adaptation constituents. Because stress-response signaling pathways are highly interconnected, many antifungal tolerance mechanisms are likely under clock control (Coca-Ruiz and Boy-Ruiz, 2025; Víglaš and Olejníková, 2021). While the exact role of *CCG-8* in clock-controlled translation remains to be fully elucidated, its likely function as a key mediator in the translational output of the clock. Our data show that the master regulators of the circadian clock - the *White Collar Complex* [*WC-2* (P78714/NCU00902), *WC-1* (Q01371/NCU02356)] and *Frequency* [P19970/NCU02265] remained unaffected under our experimental conditions. This suggests that the deletion of *CCG-8* disrupts the activity of downstream effectors, rendering the tolerance mechanisms of the cell “off-balance” without compromising conidial viability (Fig. S4).

Closely associated with ribosome biogenesis is the UPR. When cells are under stress, the UPR is activated, typically downregulating ribosome biogenesis to manage the folding and degradation of already synthesized proteins (Wimalasena et al., 2008). Supporting an altered UPR state in our model, we detected an upregulated signal for the endoplasmic reticulum chaperone, BiP (P78695/NCU03982) in conidia of *N. crassa* Δ*ccg-8* compared to the wild type. BiP ordinarily binds to Ire1 RNase; its release during stress triggers UPR activation (Krishnan and Askew, 2014).

Because we used conidia as the starting material for mycelium production, it is plausible that the Δ*ccg-8* mutant initiates growth with an attenuated UPR. This condition could consequently also lead to the observed upregulation of ribosome biogenesis (Wimalasena et al., 2008). Given that the state of the mycelium is known to affect qualities of conidia forming at the mycelium (Kunova et al., 2013), the reverse impact – where the initial state of conidia influences the proteomic profile of growing mycelium – warrants further investigation.

In our work, *N. crassa* Δ*ccg-8* exhibited increased susceptibility to various cell surface stress agents. This phenotype is consistent with proposed changes in plasma membrane, a premise supported by the *ccg-8* gene annotation (GO:0008654, phospholipid biosynthetic process) and its enrichment in a published dataset (Table 1, Table S1).

Our proteomic analysis further reinforced this link by revealing an enrichment of dysregulated proteins related to fatty acid *β*-oxidation. Although this enrichment was statistically strongest in conidia, the absolute number of fatty acid-related proteins was comparable between mycelium and conidia samples (24 and 35, respectively, Fig. 1; Table S3). Consequently, these differences pointed toward an expected alteration in the overall fatty acid composition in both samples. Indeed, we detected changes in mycelium samples (Fig. 2), key macroscopic structure evaluated during in vitro susceptibility testing. Given that fatty acids are integral constituents of phospholipids, we hypothesized that phospholipid biosynthesis would be affected. This hypothesis was also supported by the presence of individual dysregulated proteins involved in phospholipid biosynthesis (Table S2), such as Q7S5W9 (Phosphatidyl-*N*-methylethanolamine *N*-methyltransferase), F5HDG6 (Phosphatidate cytidylyltransferase), and Q1K800 (Phosphatidylserine decarboxylase proenzyme 1).

Further changes to the plasma membrane composition are supported by the dysregulation of several proteins involved in sphingolipid metabolism, including Q7S6I3 (Neutral ceramidase), Q7RXZ7 (Serine palmitoyl-transferase 2), Q7RX39 (Ceramide-binding protein *SVF-1*), and Q7SG47 (Sphingolipid long chain base-responsive protein LSP1). While changes in ceramide synthesis and degradation dynamics are known to modulate stress responses in *S. cerevisiae* (P.-W. Chen et al., 2015), the specific contribution of these dysregulated proteins to stress tolerance in *N. crassa* warrants further investigation. Regarding sterol biosynthesis, the published dataset of *CCG-8* regulated genes listed a gene NCU02624 (Table S1), which codes for lanosterol-14-*α*-demethylase (Q7SDI6/*CYP51*) – a target of azole antifungals and a key enzyme in the ergosterol pathway. Its detected dysregulation in both mycelium and conidia of *N. crassa* Δ*ccg-8* serves to validate our proteomic methodology (Table S2).

Moreover, we also detected dysregulation in other constituents of the ergosterol biosynthesis pathway: Q7S8K7 (Sterol 22-desaturase / *ERG-5*), P38670 (Δ14-sterol reductase / *ERG-24*), V5INY4 (C-4 methylsterol oxidase / *ERG-25*), and Q7S8B3 (Squalene monooxygenase / *ERG-1*). While we also detected differential signaling for Q7RYC7 (Δ14-sterol reductase / NCU04480), its incomplete UniProt annotation prevents us from definitively assigning its role in ergosterol biosynthesis (a function is fulfilled by the characterized P38670). Nevertheless, as ergosterol and sphingolipids are essential components of plasma membrane lipid rafts, this observed extensive dysregulation points to a substantial imbalance of molecules in the plasma membrane. This imbalance is the likely cause for the documented decrease in ergosterol content in the mycelium of *N. crassa* Δ*ccg-8* (Table 3).

Changes in plasma membrane components (ergosterol, phospholipids, and sphingolipids) collectively account for the increased susceptibility of Δ*ccg-8* mutant to several agents, including azoles, NaCl, sorbitol, and Tween-80. We also observed different susceptibility profile to agents that compromise cell wall integrity, such as Congo red and echinocandins, exposing the plasma membrane directly to the osmotic fluctuations of the environment. Furthermore, while the susceptibility difference was clearer at higher concentrations, the mutant displayed impaired tolerance to tunicamycin (Fig. 4), an inhibitor of *N*-glycosylation that induces endoplasmic reticulum (ER) stress (Yoon et al., 2023). This finding aligns with the role of *CCG-8* in the UPR pathway. Functionally, the Δ*ccg-8* fatty acid profile – characterized by an increase in unsaturated acids – is consistent with enhanced membrane fluidity and permeability. These structural changes likely compromise membrane integrity, which directly contributes to the increased susceptibility to azole antifungals, which rely on efficient membrane penetration. Altogether, our data strongly support a role of *CCG-8* in maintaining lipid homeostasis and membrane stability, particularly when the organism is challenged by stress conditions caused by antifungal compounds.

This is further supported by detected dysregulation of the cell wall biosynthesis enzymes: Q7SG86 (*α*-1,3-glucan synthase) and Q7S0A7 (1,3-*β*-glucan synthase) in mycelium of *N. crassa* Δ*ccg-8* (Table S2). The decreased presence of the latter enzyme in the deletion mutant supports the previously observed increased susceptibility to echinocandin antifungals.

Finally, to elaborate on the role of the Δ*ccg-8* mutation at the level of cell wall, we investigated the fungal strains using an in vivo approach: injecting *N. crassa* conidia into *Galleria mellonella* larvae. The *G. mellonella* is a suitable model for fungal infection studies due to the similarities between insect and human innate immune systems and to the comparable behaviour of pathogenic fungi in both mammals and insects. Therefore, this model is well-established for analyzing pathogens like *Candida* spp., *Cryptococcus neoformans*, and *Aspergillus fumigatus* (Giammarino et al., 2024; Wojda et al., 2020).

However, the mechanism by which *G. mellonella* larvae respond to non-pathogenic fungi like *N. crassa* remains elusive. We found that the immune system of the larvae clearly distinguished between conidia of *N. crassa* wtA (wild type) and *N. crassa* Δ*ccg-8*. Macroscopic (Fig. S2) and microscopic (Fig. 9, Fig. 10) analyses showed that the conidia of Δ*ccg-8* mutant were cleared from the larvae faster. We initially expected differences in surface sugar composition to be the determining factor, but only slight changes were observed in conidia (Fig. 3). On the other hand, FTIR analysis revealed notable changes on mycelium level. The quantitative curve-fitting revealed shifts in v_as_(CH_2_)/v_as_(CH_3_) and v_s_(CH_2_)/v_s_(CH_3_) ratios (corroborating observed lipidomic differences) as well as changes at the cell surface, i.e., difference in protein-to-glycan ratio pointing to different protein composition of *N. crassa* Δ*ccg-8*. The proteomic data provided an interesting insight in the surface of conidia (Fig. 1D). The differential log2 mean signals indicate a marked downregulation of specific cell-surface proteins in *Δccg-8* conidia. This reduction in protein coverage likely facilitates more rapid recognition of underlying cell-wall saccharides by the larval immune system. A key example of this downregulation includes hydrophobin/EAS/*CCG-2* protein (Q04571/NCU08457), coded by another clock-controlled gene, the expression of which was probably disrupted by deletion of the *CCG-8* transcription factor.

This conclusion is consistent with the in vivo analysis showing accelerated clearing of mutant conidia, suggesting there is still a possibility of subtle surface distinctions between conidia of tested strains. To definitively confirm these surface alterations, future studies should employ direct methods, such as antibodies targeting cell wall glycoproteins (e.g., via resources like CarboSource). Furthermore, this research could be expanded beyond *N. crassa* Δ*ccg-8* by leveraging the comprehensive collection of available deletion mutants. Correlating these molecular surface profiles with in vivo experiments in larvae would significantly enhance the robustness of cell surface characterization.

In contrast to the non-pathogens, the pathogen *A. fumigatus* CCF6600 survived the effects of immune system of *G. mellonella*. The survival profile between the pathogens and non-pathogens (Fig. 7) suggests that the mortality of larvae was in the case of pathogens caused by the pathogenic activity of *A. fumigatus* CCF6600. The conidia of *N. crassa* were already not present in the bodies of larvae, when their survival dropped. This suggests that the mortality associated with *N. crassa* was likely due to damage from intense internal clearing processes (i.e., “inflammation-like processes”) rather than infection (Smith and Casadevall, 2021). Yet, further clarification on the difference in reaction of *G. mellonella* to the proteins that are exposed to the environment/immune system at the surface of non-pathogens remains for future studies. In summary, we demonstrated that the *G. mellonella* model can be successfully applied to study both pathogenic and non-pathogenic filamentous fungi. This broadens the utility of *N. crassa* as a reference fungus while providing a comprehensive characterization of the Δ*ccg-8* deletion mutant in the context of antifungal resistance and in vivo host interactions.

## 5 Conclusions

This work demonstrates that the deletion of the *ccg-8* gene, which provides basal tolerance to ketoconazole, impacts *N. crassa* on a much broader level by interfering with fundamental cell surface structures and metabolic homeostasis. The Δ*ccg-8* mutation caused dysregulation of proteins across several key pathways, notably lipid metabolism and ribosome biogenesis. The latter reflecting the clock-controlled nature of the *CCG-8* transcription factor. Alterations in lipid metabolism were corroborated by direct fatty acid determination and a significant reduction in ergosterol content. Together with proteomic changes in the sphingolipid biosynthesis, the results substantiated the observed increased susceptibility of *N. crassa* Δ*ccg-8* to various stress agents, including azoles. In terms of susceptibility to echinocandins, the picture is completed by dysregulation of *β-*glucan synthase (target enzyme). Furthermore, glycomic and proteomic analyses confirm that the mutant mycelium possesses a distinct surface protein composition compared to the wild type. While surface alterations in conidia were more subtle, they correlated with the observed in vivo results: the non-pathogenic *N. crassa* Δ*ccg-8* conidia were rapidly and effectively recognized and cleared by the *G. mellonella* immune system. The capability of studying *N. crassa* in an in vivo model opens a new area of research, where results on non-pathogens can be compared with effects exerted by pathogens, and so accelerating research on the interaction of fungi with the environment, for example, in the area of antifungal resistance and tolerance.

## Declaration of generative AI use

During the preparation of this work the authors used Google Gemini in order to refine fluency, coherence and cohesion of the text of the article and check for grammatical errors. After using this tool/service, the authors reviewed and edited the content as needed and takes full responsibility for the content of the published article.

## CRediT authorship contribution statement

**Ján Víglaš:** Writing – original draft, Visualization, Supervision, Project administration, Methodology, Investigation, Funding acquisition, Formal analysis, Conceptualization. **Katarína Víglaš:** Writing – review & editing, Writing – original draft, Resources, Methodology, Investigation, Formal analysis. **Pavol Farkaš:** Writing – review & editing, Writing – original draft, Resources, Methodology, Investigation, Formal analysis. **Jana Bellová:** Writing – original draft, Resources, Investigation, Formal analysis. **Peter Baráth:** Writing – original draft, Resources, Methodology, Investigation, Formal analysis, Data curation. **Peter Gajdoš:** Writing – review & editing, Resources, Investigation, Formal analysis. **Tatiana Klempová:** Writing – review & editing, Resources, Investigation, Formal analysis. **Boris Lakatoš:** Writing – review & editing, Validation, Methodology. **Petra Olejníková:** Writing – review & editing, Writing – original draft, Validation, Resources, Investigation, Formal analysis.

## Funding

This work was Funded by the EU NextGenerationEU through the Recovery and Resilience Plan for Slovakia under the project No. 09I03-03-V04-00659.

## Declaration of competing interest

The authors declare that they have no known competing financial interests or personal relationships that could have appeared to influence the work reported in this paper.

## Data Availability

The proteomic data were deposited to the ProteomeXchange Consortium via the PRIDE (Perez-Riverol et al., 2025) partner repository with the dataset identifier PXD070373 and DOI: 10.6019/PXD070373.

## Appendix A Supplementary data

Supplementary material 1 Table S1 - GO enrichment analysis for transcriptomic dataset of *Neurospora crassa* Δ*ccg-8* first presented in Hu et al. (2018). Image 4

Supplementary material 2 Table S2 - Quantified proteomic profiles (including GO enrichment analysis) of *Neurospora crassa* wild-type and *ccg-8* strains obtained in our study. Image 5

Supplementary material 3 Table S3 - Detailed proteomic data for highlighted proteins in Figure 1. Image 6

Supplementary material 4 Figure S1-S6, Table S4 - Phenotypic characterization and biochemical analysis of *N. crassa Δccg-8*. Image 7

## References

[bb0005] Adt I., Toubas D., Pinon J.-M., Manfait M., Sockalingum G.D. (2006). FTIR spectroscopy as a potential tool to analyse structural modifications during morphogenesis of Candida albicans. Arch. Microbiol..

[bb0010] Berman J., Krysan D.J. (2020). Drug resistance and tolerance in fungi. Nat. Rev. Microbiol..

[bb0015] Bikmurzin R., Bandzevičiūtė R., Maršalka A., Maneikis A., Kalėdienė L. (2022). FT-IR method limitations for β-glucan analysis. Molecules.

[bb0020] Bzducha-Wróbel A., Farkaš P., Bieliková S., Čížová A., Sujkowska-Rybkowska M. (2024). How do the carbon and nitrogen sources affect the synthesis of β-(1,3/1,6)-glucan, its structure and the susceptibility of Candida utilis yeast cells to immunolabelling with β-(1,3)-glucan monoclonal antibodies?. Microb. Cell Factories.

[bb0025] Certik M., Shimizu S. (2000). Kinetic analysis of oil biosynthesis by an arachidonic acid-producing fungus, Mortierella alpina 1S-4. Appl. Microbiol. Biotechnol..

[bb0030] Chen P.-W., Fonseca L.L., Hannun Y.A., Voit E.O. (2015). Dynamics of the heat stress response of ceramides with different fatty-acyl chain lengths in baker’s yeast. PLoS Comput. Biol..

[bb0035] Chen X., Xue W., Zhou J., Zhang Z., Wei S., Liu X., Sun X., Wang W., Li S. (2016). De-repression of CSP-1 activates adaptive responses to antifungal azoles. Sci. Rep..

[bb0040] Coca-Ruiz, V., & Boy-Ruiz, D. (2025). Timing is everything: the fungal circadian clock as a master regulator of stress response and pathogenesis. In *Stresses* (vol. 5, number 3, p. 47). Multidisciplinary Digital Publishing Institute (MDPI). doi:10.3390/stresses5030047.

[bb0045] Colot H.V., Park G., Turner G.E., Ringelberg C., Crew C.M., Litvinkova L., Weiss R.L., Borkovich K.A., Dunlap J.C. (2006). A high-throughput gene knockout procedure for neurospora reveals functions for multiple transcription factors. Proc. Natl. Acad. Sci..

[bb0050] Cox J., Hein M.Y., Luber C.A., Paron I., Nagaraj N., Mann M. (2014). Accurate proteome-wide label-free quantification by delayed normalization and maximal peptide ratio extraction, termed MaxLFQ*. Mol. Cell. Proteomics.

[bb0055] Ding Z., Lamb T.M., Boukhris A., Porter R., Bell-Pedersen D. (2021). Circadian clock control of translation initiation factor eIF2α activity requires eIF2γ-dependent recruitment of rhythmic PPP-1 phosphatase in neurospora crassa. MBio.

[bb0060] Dong W., Tang X., Yu Y., Nilsen R., Kim R., Griffith J., Arnold J., Schüttler H.-B. (2008). Systems biology of the clock in Neurospora crassa. PLoS One.

[bb0065] Dróżdż A., Kubera D., Olender A., Dabrowski W., Szukala M., Wosko S., Chwiej J., Rugiel M., Kawoń K., Gagoś M. (2025). ATR-FTIR spectroscopic markers indicating drug resistance in selected Candida strains. Sci. Rep..

[bb0070] Giammarino, A., Bellucci, N., & Angiolella, L. (2024). *Galleria mellonella* as a model for the study of fungal pathogens: advantages and disadvantages. In *Pathogens* (vol. 13, number 3, p. 233). Multidisciplinary Digital Publishing Institute (MDPI). doi:10.3390/pathogens13030233. PMC10976154 38535576

[bb0075] Gu X., Xue W., Yin Y., Liu H., Li S., Sun X. (2016). The Hsp90 co-chaperones Sti1, Aha1, and P23 regulate adaptive responses to antifungal azoles. Front. Microbiol..

[bb0080] Havlik D., Brandt U., Bohle K., Fleißner A. (2017). Establishment of neurospora crassa as a host for heterologous protein production using a human antibody fragment as a model product. Microb. Cell Factories.

[bb0085] Hsu L.C., Wang S.L., Lin Y.C., Wang M.K., Chiang P.N., Liu J.C., Kuan W.H., Chen C.C., Tzou Y.M. (2010). Cr(VI) removal on fungal biomass of neurospora crassa: the importance of dissolved organic carbons derived from the biomass to Cr(VI) reduction. Environ. Sci. Technol..

[bb0090] Hu Y., Qin Y., Liu G. (2018). Collection and curation of transcriptional regulatory interactions in Aspergillus nidulans and Neurospora crassa reveal structural and evolutionary features of the regulatory networks. Front. Microbiol..

[bb0095] Hughes C.S., Moggridge S., Müller T., Sorensen P.H., Morin G.B., Krijgsveld J. (2019). Single-pot, solid-phase-enhanced sample preparation for proteomics experiments. Nat. Protoc..

[bb0100] Krishnan K., Askew D.S. (2014). Endoplasmic reticulum stress and fungal pathogenesis. Fungal Biol. Rev..

[bb0105] Kunova A., Pizzatti C., Cortesi P. (2013). Impact of tricyclazole and azoxystrobin on growth, sporulation and secondary infection of the rice blast fungus, Magnaporthe oryzae. Pest Manag. Sci..

[bb0110] Machová E., Bystrický S. (2012). Yeast mannans protect liposomes against peroxidation but do not scavenge free radicals. Carbohydr. Polym..

[bb0115] Novák M., Synytsya A., Gedeon O., Slepička P., Procházka V., Synytsya A., Blahovec J., Hejlová A., Čopíková J. (2012). Yeast β(1–3),(1–6)-d-glucan films: preparation and characterization of some structural and physical properties. Carbohydr. Polym..

[bb0120] Patel, P. K., & Free, S. J. (2019). The genetics and biochemistry of cell wall structure and synthesis in *Neurospora crassa*, a model filamentous fungus. In *Front. Microbiol.* (vol. 10, p. 2294). doi:10.3389/fmicb.2019.02294. PMC6796803 31649638

[bb0125] Pereira T.C., De Barros P.P., Fugisaki L.R., Rossoni R.D., Ribeiro F.D., De Menezes R.T., Junqueira J.C., Scorzoni L. (2018). Recent advances in the use of *Galleria mellonella* model to study immune responses against human pathogens. J. Fungi.

[bb0130] Perez-Riverol Y., Bandla C., Kundu D.J., Kamatchinathan S., Bai J., Hewapathirana S., John N.S., Prakash A., Walzer M., Wang S., Vizcaíno J.A. (2025). The PRIDE database at 20 years: 2025 update. Nucleic Acids Res..

[bb0135] Robbins N., Caplan T., Cowen L.E. (2017). Molecular evolution of antifungal drug resistance. Ann. Rev. Microbiol..

[bb0140] Segéňová I., Víglaš J., Pagáč T., Olejníková P. (2025). Adaptation under pressure: resistance and stress response interplay in clinical Aspergillus fumigatus isolates. J. Fungi.

[bb0145] Sheehan G., Clarke G., Kavanagh K. (2018). Characterisation of the cellular and proteomic response of Galleria mellonella larvae to the development of invasive aspergillosis. BMC Microbiol..

[bb0150] Slaný O., Klempová T., Cibulková Z., Marcinčák S., Shapaval V., Čertík M. (2025). Evaluation of stability and quality of bioproducts derived from solid-state fermentation of wheat bran using Mortierella alpina. J. Food Sci..

[bb0155] Smith F.Q., Casadevall A. (2021). Fungal immunity and pathogenesis in mammals versus the invertebrate model organism *Galleria mellonella*. Pathog. Dis..

[bb0160] Sun X., Wang K., Yu X., Liu J., Zhang H., Zhou F., Xie B., Li S. (2014). Transcription factor CCG-8 as a new regulator in the adaptation to antifungal azole stress. Antimicrob. Agents Chemother..

[bb0165] Verdín J., Sánchez-León E., Rico-Ramírez A.M., Martínez-Núñez L., Fajardo-Somera R.A., Riquelme M. (2019). Off the wall: the rhyme and reason of Neurospora crassa hyphal morphogenesis. The Cell Surface.

[bb0170] Víglaš J., Olejníková P. (2021). Signalling mechanisms involved in stress response to antifungal drugs. Res. Microbiol..

[bb0175] Víglaš J., Olejníková P. (2023). Antifungal azoles trigger a xenobiotic detoxification pathway and chitin synthesis in Neurospora crassa. Res. Microbiol..

[bb0180] Vogel H.J. (1956). A convenient growth medium for Neurospora (Medium N). Microbial Gen. Bull..

[bb0185] Wang K., Zhang Z., Chen X., Sun X., Jin C., Liu H., Lia S. (2015). Transcription factor ADS-4 regulates adaptive responses and resistance to antifungal azole stress. Antimicrob. Agents Chemother..

[bb0190] Weichert M., Herzog S., Robson S.A., Brandt R., Priegnitz B.E., Brandt U., Schulz S., Fleißner A. (2020). Plasma membrane fusion is specifically impacted by the molecular structure of membrane sterols during vegetative development of Neurospora crassa. Genetics.

[bb0195] WHO (2022). WHO fungal priority pathogens list to guide research, development and public health action. https://iris.who.int/bitstream/handle/10665/363682/9789240060241-eng.pdf?sequence=1.

[bb0200] Wimalasena T.T., Enjalbert B., Guillemette T., Plumridge A., Budge S., Yin Z., Brown A.J.P., Archer D.B. (2008). Impact of the unfolded protein response upon genome-wide expression patterns, and the role of Hac1 in the polarized growth, of Candida albicans. Fungal Genet. Biol..

[bb0205] Wojda, I., Staniec, B., Sułek, M., & Kordaczuk, J. (2020). The greater wax moth *galleria mellonella*: biology and use in immune studies. In Pathog. Dis. (Vol. vol. 78, Number 9). Oxford Academic. doi:10.1093/femspd/ftaa057. PMC7683414 32970818

[bb0210] Xue W., Yin Y., Ismail F., Hu C., Zhou M., Cao X., Li S., Sun X. (2019). Transcription factor CCG-8 plays a pivotal role in azole adaptive responses of neurospora crassa by regulating intracellular azole accumulation. Curr. Genet..

[bb0215] Yin Y., Zhang H., Zhang Y., Hu C., Sun X., Liu W., Li S. (2021). Fungal Zn(II)2Cys6 transcription factor ADS-1 regulates drug efflux and ergosterol metabolism under antifungal azole stress. Antimicrob. Agents Chemother..

[bb0220] Yoon D., Moon J.H., Cho A., Boo H., Cha J.S., Lee Y., Yoo J. (2023). Structure-based insight on the mechanism of N-glycosylation inhibition by tunicamycin. Mol. Cell.

[bb0225] Zhou M., Hu C., Yin Y., Wang J., Ye S., Yu Y., Sun X., Li S. (2022). Experimental evolution of multidrug resistance in neurospora crassa under antifungal azole stress. J. Fungi.

