# Supplementary material for: Neurospora crassa Δccg-8 compromises cell surface integrity and antifungal tolerance: Insights from in vitro and Galleria mellonella studies
Source: Cell Surf. 2026 Mar 19;15:100172. doi: 10.1016/j.tcsw.2026.100172 (PMC13053782; doi:10.1016/j.tcsw.2026.100172)
Supplement: Supplementary material 4 — Figure S1-S6, Table S4 - Phenotypic characterization and biochemical analysis of N. crassa Δccg-8. [file mmc4.docx]

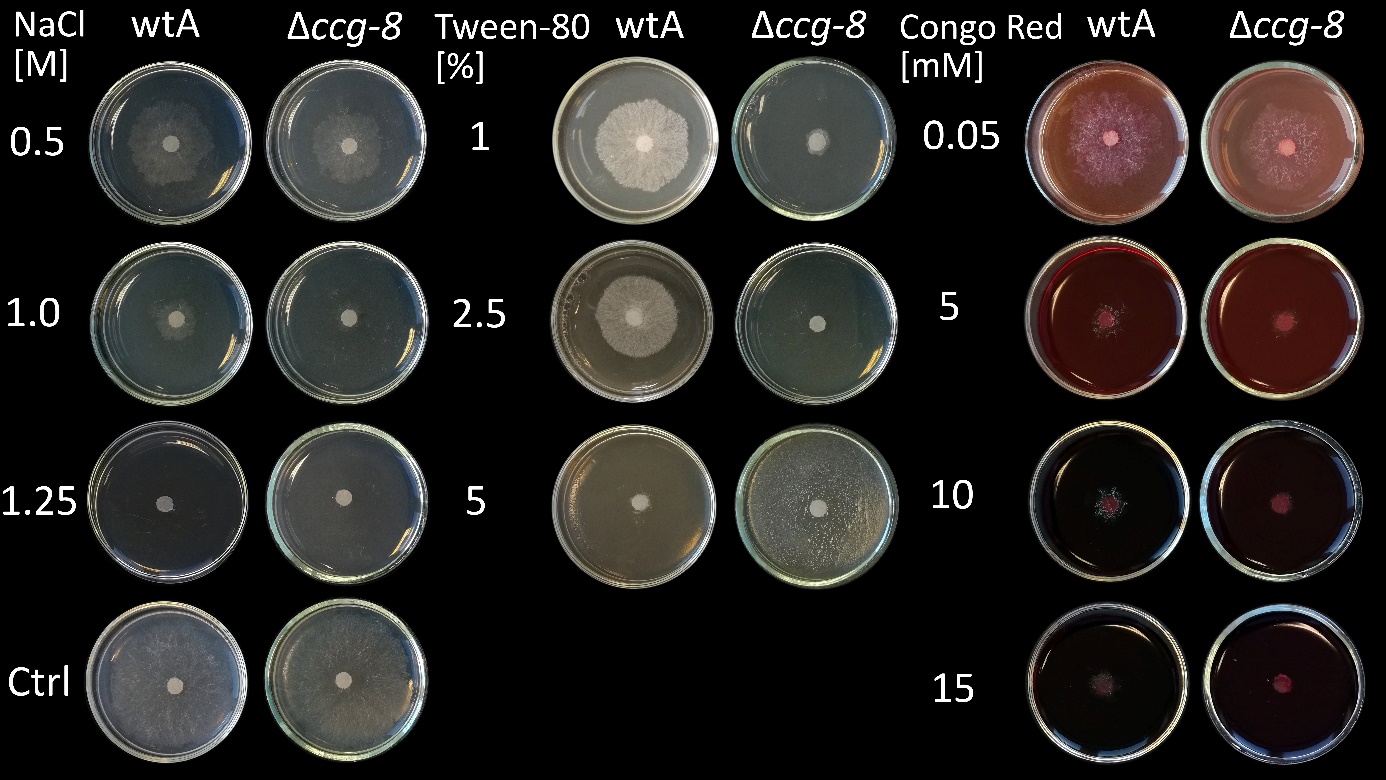


Figure S1. The effect of different cell surface stressors on the growth of *N. crassa* Δ*ccg-8* compared to wild type strain “wt”. Ctrl = control without a stressor. Concentrations indicated are of compounds present in the cultivation medium (for Tween-80 it is % *v*/*v*).


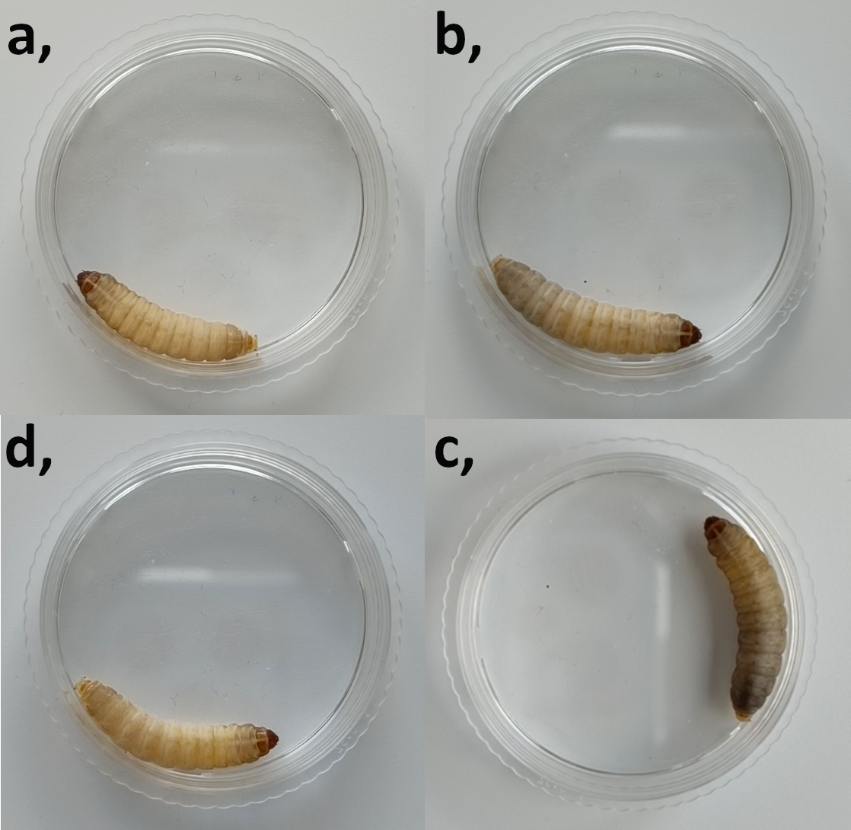


Figure S2. The reaction of *G. mellonella* to the conidia of tested fungi, 7 min post-injection. a, injected by Control – PBS+Tween-80; b, *N. crassa* wtA. c, *N. crassa* Δ*ccg-8* (both strains at dose 1 × 10^6^ conidia per larva) d, *Aspergillus fumigatus* CCF6600 - positive control - pathogenic model (dose 5 × 10^5^ conidia per larva). *G. mellonella* injected with *N. crassa* Δ*ccg-8* is the most melanized, followed by larvae with *N. crassa* wtA and then infected larvae with *A. fumigatus* are comparable to control.


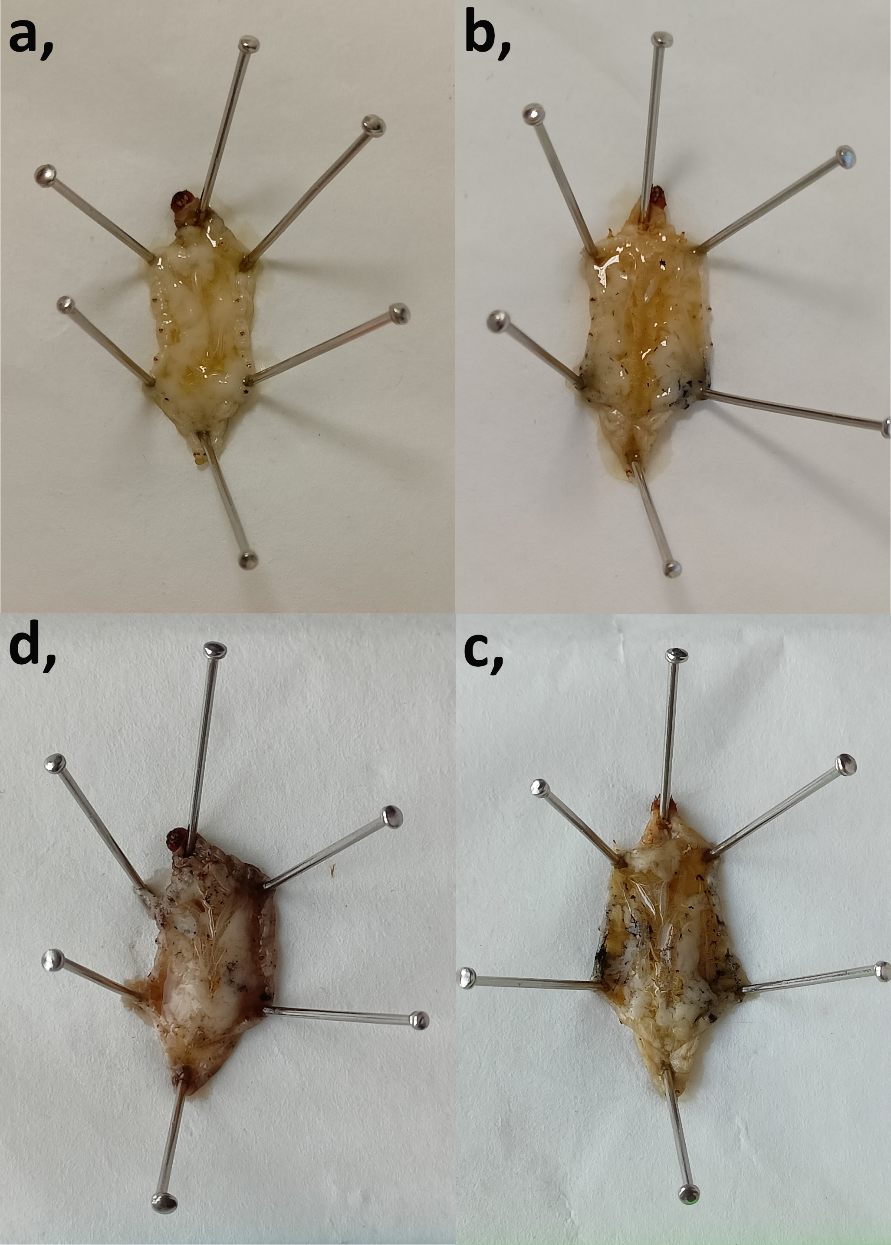


Figure S3. The dissection of *G. mellonella* 48 h post-injection with the conidia (dose 1 × 10^5^ per larva) of tested fungi: a, injected by Control – PBS+Tween-80; b, *N. crassa* wtA. c, *N. crassa* Δ*ccg-8*, d, *Aspergillus fumigatus* CCF6600. Black granule/flocule-like structures (nodules) were removed and analyzed (Figures 6, 7 and 8). *N. crassa Δccg-8* (c) – the most abundant nodules, *A. fumigatus* CCF6600 – more spherical nodules


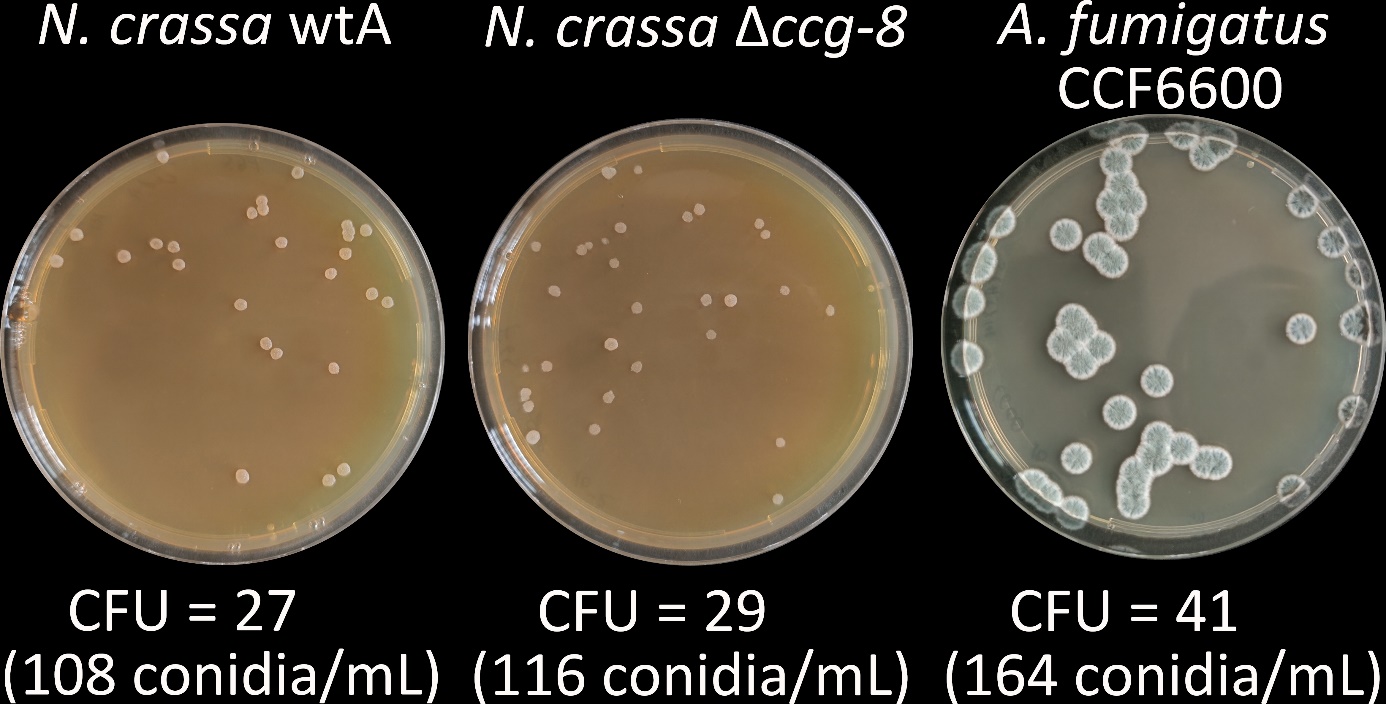


Figure S4. Assessment of conidial viability prior to larval injection. The spore suspensions were adjusted to 100 conidia/mL (determined by direct counting), followed by plating 250 µL onto colony-inducing media. Expected CFU = 25. Variations in observed CFU for *A. fumigatus* are attributed to the technical difficulty of enumerating its smaller conidia (data not shown). The viability constitutes practically 100%


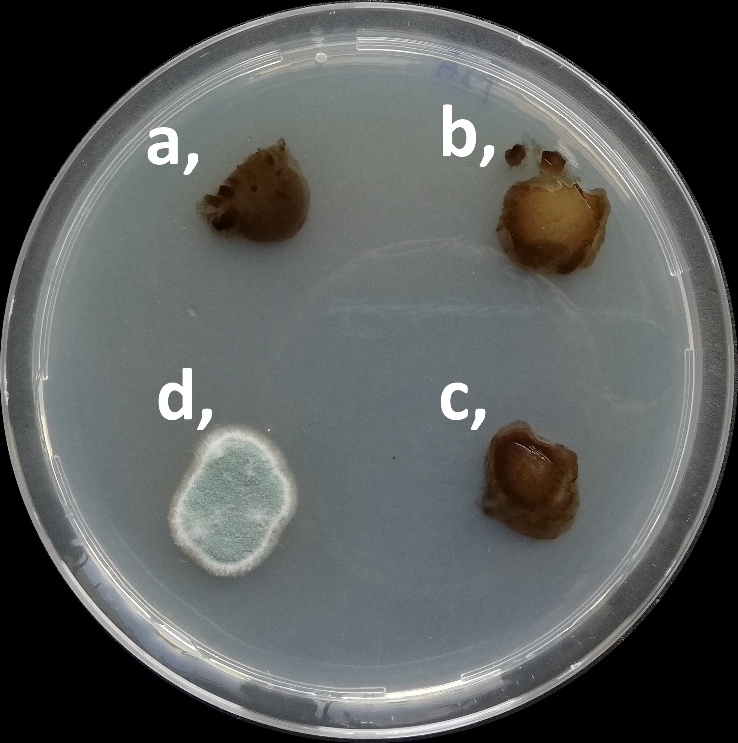


Figure S5. The growth of tested fungi after removal from the bodies of *G. mellonella* 48 h post-injection. The body of larvae was open and pressed upon fresh PDA agar, followed by cultivation at 30 °C for 48 hours. a, injected by Control – PBS+Tween-80; b, *N. crassa* wtA. c, *N. crassa* Δ*ccg-8*, d, *Aspergillus fumigatus* CCF6600. Only *A. fumigatus* CCF6600 is growing. Conidia of *N. crassa* were cleared at this point out of the body of larvae by their immune system (Figures 8, 9).

*N. crassa* wtA

*N. crassa* Δ*ccg-8*

Figure S6. Curve-ﬁtting of FTIR spectra of the mycelia “whole cell” measurement, region. Identified “area under curve” values are displayed in Table S4.

Table S4 a. Identified absorption bands (wavenumbers in cm^–1^) from second-derivative ATR-FTIR spectra after curve-fitting of *N*. *crassa* „whole cell“ samples [A/a.u.] stands for area under curve in arbitrary units

| **This work** | | | | | | | | **Hsu et al., 2010** | **Dróżdż et al., 2025** | | |
| --- | --- | --- | --- | --- | --- | --- | --- | --- | --- | --- | --- |
| **Mycelium, wtA** | | **Mycelium, Δccg-8** | | **Conidia, wtA** | | **Conidia, Δccg-8** | | ***N. crassa*** | ***C. albicans*** | **Assignment** | **Origin** |
| ***ν*/cm^–1^** | ***A*/a.u.** | ***ν*/cm^–1^** | ***A*/a.u.** | ***ν*/cm^–1^** | ***A*/a.u.** | ***ν*/cm^–1^** | ***A*/a.u.** | ***ν*/cm^–1^** | |  |  |
| **3265** |  | **3066** |  | **3272** |  | **3277** |  |  |  | *ν*(N–H); *ν*(O–H) | Protein, polysaccharide |
| **2960** | **0.7** | **2958** | **0.5** | **2966** | **0.9** | **2962** | **0.7** |  | **2958** | *ν*_as_(CH_3_) | Lipids, (CH_2_OH in sugars) |
| **2932** | **3.8** | **2928** | **3.0** | **2926** | **2.8** | **2926** | **2.5** | **2926** | **2918** | *ν*_as_(CH_2_) |  |
| **2876** | **1.1** | **2871** | **1.9** | **2874** | **0.9** | **2874** | **0.8** |  | **2873** | *ν*_s_(CH_3_) |  |
| **2852** | **1.1** | **2850** | **1.0** | **2852** | **0.5** | **2852** | **0.4** | **2855** | **2850** | *ν*_s_(CH_2_) |  |
| **1745** | **0.7** | **–** | **–** | **1734** | **0.3** | **1734** | **0.3** | **1737** | **1745** | *ν*(C=O) | Lipids, phospholipids (esters) |
| **1710** | **1.1** | **1713** | **0.3** | **–** | **–** | **–** | **–** |  | **1716** |  |  |
| **1682** | **2.5** | **1674** | **3.7** | **1682** | **1.6** | **1682** | **1.4** |  | **1683** | *ν*(C=O), *δ*(NH) | Amide I, proteins, ap. *β*-sheet and *β*-turn |
| **1646** | **7.3** | **–** | **–** | **1650** | **5.6** | **–** | **–** | **1655** | **1655** |  | Amide I, proteins, *α*-helix, chitin |
| **1630** | **10.1** | **1629** | **6.8** | **1630** | **13.7** | **1638** | **20.4** |  | **1635** |  | Amide I, proteins, *β*-sheet |
| **1597** | **1.1** | **1607** | **2.0** | **–** | **–** | **1594** | **0.5** |  |  |  | Amide I, proteins |
| **1572** | **1.1** | **1580** | **16.3** | **1583** | **2.5** | **1575** | **1.3** |  |  | *δ*(NH), *ν*(CN) | Amide II, proteins |
| **1546** | **5.0** | **–** | **–** | **1544** | **8.2** | **1542** | **8.0** | **1550** | **1545** |  |  |
| **1517** | **1.4** | **1515** | **6.8** | **1516** | **3.0** | **1516** | **3.3** |  | **1515** |  |  |
| **1495** | **0.0** | **–** | **–** | **1496** | **0.0** | **–** | **–** |  | **1495** |  |  |
| **–** | **–** | **–** | **–** | **–** | **–** | **–** | **–** |  | **1467** | *δ*(CH_2_), δ(CH_3_) | Lipids, proteins |
| **1460** | **3.1** | **1458** | **1.6** | **1453** | **2.4** | **1453** | **2.6** |  | **1454** |  |  |
| **–** | **–** | **–** | **–** | **–** | **–** | **–** | **–** |  | **1438** |  |  |
| **1413** | **3.5** | **–** | **–** | **–** | **–** | **–** | **–** |  | **1413** |  |  |
| **–** | **–** | **1404** | **5.4** | **1399** | **3.9** | **1399** | **3.9** |  | **1402** |  |  |
| **1372** | **2.6** | **–** | **–** | **1377** | **1.7** | **1372** | **1.5** | **1383** | **1368** |  |  |
| **1343** | **1.1** | **1351** | **5.4** | **1343** | **1.0** | **1343** | **1.1** |  | **1342** | *δ*(CH_2_) | Phospholipids, amino acids |
| **–** | **–** | **1329** | **1.8** | **–** | **–** | **–** | **–** |  |  | *δ*(NH) | Amide III, proteins |
| **1311** | **3.2** | **1315** | **1.7** | **1309** | **2.8** | **1309** | **2.5** |  | **1300** |  |  |
| **1242** | **4.8** | **1228** | **1.3** | **1238** | **5.1** | **1238** | **5.0** |  | **1240** |  |  |
| **–** | **–** | **–** | **–** | **–** | **–** | **–** | **–** |  | **1230** |  |  |
| **1202** | **0.4** | **1191** | **0.2** | **1203** | **0.6** | **1203** | **0.7** |  | **1201** | *ν*_as_(PO_2_^–^) | Phosphomannan, polysaccharide |
| **1150** | **3.3** | **1150** | **2.5** | **1150** | **1.9** | **1150** | **2.1** | **1152** | **1155** | *ν*(COC) | *β*-1,3-Glucans |
| **–** | **–** | **–** | **–** | **–** | **–** | **–** | **–** |  | **1125** | *ν*(CO), *ν*(CC), *ν*(COC) | α-Glucans, RNA |
| **1109** | **4.4** | **1107** | **3.3** | **1110** | **4.9** | **1109** | **4.3** |  | **1105** | *ν*(CO), *ν*(CC), δ(COH) | Glycogen, *β*-1,3-glucans |
| **1081** | **6.1** | **1077** | **6.2** | **1081** | **3.1** | **1080** | **3.8** | **1077** | **1077** | *ν*s(PO2–)*, ν*(CO), *ν*(OH) | Glycogen, phosphomannan, DNA |
| **1038** | **26.7** | **1054** | **4.4** | **1035** | **27.8** | **1035** | **29.3** | **1040** | **1043** | *ν*(CO), *ν*(OH) | Glycogen, mannans |
| **–** | **–** | **1028** | **20.6** | **–** | **–** | **–** | **–** |  | **1021** | *δ*(CO) | Glycogen |
| **996** | **2.3** | **983** | **2.2** | **991** | **2.9** | **991** | **1.9** |  | **996** | *ν*(CC), *δ*(CO) | *β*-1,6-Glucans |
| **966** | **0.3** | **945** | **0.0** | **966** | **0.4** | **965** | **0.3** |  | **966** |  | Mannans |
| **927** | **0.9** | **921** | **0.9** | **927** | **1.9** | **928** | **1.4** |  | **919** |  | Mannans |
| **892** |  | **896** |  | **879** |  | **892** |  |  | **885** |  | Glucans, mannans |
| **862** |  | **868** |  | **–** |  | **860** |  |  |  |  |  |
| **–** |  | **–** |  | **854** |  | **–** |  |  |  | *δ*(CH) | Glucans |
| **811** |  | **819** |  | **805** |  | **806** |  |  | **809** |  |  |
| **780** |  | **773** |  | **773** |  | **778** |  |  | **779** |  |  |
| **762** |  | **–** |  | **–** |  | **761** |  |  | **762** |  |  |
| **718** |  | **–** |  | **–** |  | **–** |  |  | **719** |  |  |

**Pearson correlation coefficient**

**Mycelium wtA to Δ*ccg-8 r* = 0.16**

***r^2^* = 0.10**

**Conidia wtA to Δ*ccg-8 r* = 0.98**

***r^2^* = 0.96**

Table S4 b. Identified absorption bands (wavenumbers in cm^–1^) from second-derivative ATR-FTIR spectra after curve-fitting of *N*. *crassa* glucan samples (mycelium analysis). [A/a.u.] stands for area under curve in arbitrary units

| **wtA** | | **Δccg-8** | | **Assignment** | **Origin** |
| --- | --- | --- | --- | --- | --- |
| ***ν*/cm^–1^** | ***A*/a.u.** | ***ν*/cm^–1^** | ***A*/a.u.** |  |  |
| **3296** | **–** | **3296** | **–** | ν(N–H); ν(O–H) | Proteins, polysaccharides |
| **2958** | **0.4** | **2960** | **0.6** | *ν*_as_(CH_3_) | Lipids, (CH_2_OH in sugars) |
| **2925** | **2.1** | **2922** | **2.2** | *ν*_as_(CH_2_) |  |
| **2884** | **2.2** | **2876** | **1.2** | *ν*_s_(CH_3_) |  |
| **2850** | **0.1** | **2852** | **0.7** | *ν*_s_(CH_2_) |  |
| **1747** | **0.2** | **1739** | **0.9** | ν(C=O) | Lipids, phospholipids (esters) |
| **1699** | **0.1** | **–** | **–** |  |  |
| **1657** | **1.7** | **1654** | **1.5** | *ν*(C=O), *δ*(NH) | Amide I, proteins (*α*-helix) |
| **1625** | **1.5** | **1625** | **2.2** |  | Amide I, proteins (*β*-sheet) |
| **1558** | **1.4** | **1557** | **1.9** | δ(NH), ν(CN) | Amide II, proteins |
| **1522** | **1.0** | **1519** | **0.0** |  | Amide II, proteins, tyrosine |
| **1459** | **1.9** | **1458** | **1.7** | δ(CH2), δ(CH3) | Lipids, proteins |
| **1413** | **3.2** | **1418** | **2.6** |  |  |
| **1371** | **6.4** | **1372** | **4.4** |  |  |
| **1305** | **5.0** | **1311** | **3.1** | δ(NH) | Amide III, proteins |
| **1251** | **2.7** | **1252** | **1.6** |  |  |
| **1231** | **0.0** | **1231** | **1.5** |  |  |
| **1201** | **0.7** | **1202** | **1.0** | νas(PO2–) | Phosphomannans, polysaccharides |
| **1152** | **3.5** | **1153** | **4.7** | ν(COC) | *β*-1,3-Glucans |
| **1109** | **4.9** | **1110** | **6.1** | ν(CO), ν(CC), δ(COH) | Glycogen, *β*-1,3-Glucans |
| **1078** | **5.9** | **1077** | **6.5** | ν(CO), ν(OH) | Glycogen, phosphomannan, DNA |
| **1019** | **39.8** | **1029** | **39.1** | δ(CO) | Glucans |
| **989** | **13.3** | **990** | **13.2** | ν(CC), δ(CO) | *β*-1,6-Glucans |
| **926** | **1.8** | **924** | **3.5** |  | Mannans, glucans |
| **891** | **–** | **891** | **–** |  | *β*-Glucans |
| **844** | **–** | **851** | **–** | δ(CH) | *α*-Configuration |
| **760** | **–** | **760** | **–** |  | *α*-Glucans |
| **709** | **–** | **711** | **–** |  | *α*-Glucans |

| **Pearson corellation coefficient, *r* =** | **0.992** |
| --- | --- |
| ***r*^2^ =** | **0.985** |

Table S4 c. Identified absorption bands (wavenumbers in cm^–1^) from second-derivative ATR-FTIR spectra after curve-fitting of *N*. *crassa* glycoprotein samples (mycelium analysis). [A/a.u.] stands for area under curve in arbitrary units

| **wtA** | | **Δccg-8** | | **Assignment** | **Origin** |
| --- | --- | --- | --- | --- | --- |
| ***ν*/cm^–1^** | ***A*/a.u.** | ***ν*/cm^–1^** | ***A*/a.u.** |  |  |
| **3295** |  | **3282** |  | *ν*(N–H); *ν*(O–H) | Protein, polysaccharide |
| **2962** | **0.4** | **2963** | **0.4** | *ν*_as_(CH_3_) | Lipids, (CH_2_OH in sugars) |
| **2926** | **2.1** | **2931** | **1.5** | *ν*_as_(CH_2_) |  |
| **2876** | **0.8** | **2874** | **1.1** | *ν*_s_(CH_3_) |  |
| **2851** | **0.3** | **2851** | **0.1** | *ν*_s_(CH_2_) |  |
| **1735** | **0.2** | **1733** | **0.0** | *ν*(C=O) | Lipids, phospholipids (esters) |
| **–** | **–** | **1681** | **0.2** | *ν*(C=O), *δ*(NH) | Amide I, proteins |
| **1648** | **17.9** | **1648** | **28.3** |  | Amide I, proteins (*β*-sheet) |
| **1588** | **1.8** | **–** | **–** | *δ*(NH), *ν*(CN) | Amide II, proteins |
| **1538** | **5.0** | **1538** | **4.8** |  | Amide II, proteins (antiparallel *β*-sheet) |
| **1518** | **1.6** | **1519** | **2.5** |  | Amide II, proteins, tyrosine |
| **1463** | **0.3** | **1468** | **0.2** | *δ*(CH2), *δ*(CH3) | Lipids, proteins |
| **1455** | **0.9** | **1454** | **0.6** |  |  |
| **–** | **–** | **1435** | **0.4** |  |  |
| **1411** | **2.6** | **1410** | **1.1** |  |  |
| **–** | **–** | **1395** | **1.2** |  |  |
| **1371** | **1.8** | **1372** | **1.1** |  |  |
| **1337** | **1.2** | **1338** | **0.5** | *δ*(CH_2_) | Phospholipids and amino acids |
| **1248** | **4.9** | **1249** | **3.9** | *δ*(NH) | Amide III, proteins |
| **1210** | **1.3** | **1216** | **2.8** |  |  |
| **1152** | **2.1** | **1151** | **1.6** | *ν*(COC) | *β*-1,3-Glucans |
| **1118** | **3.6** | **1119** | **2.1** | *ν*(CO), *ν*(CC), *ν*(COC) | α-Glucans |
| **1079** | **6.2** | **1076** | **14.2** | *ν*(CO), *ν*(OH) | Glycogen, phosphomannan, DNA |
| **1042** | **13.4** | **1039** | **7.1** | *ν*(CO), *ν*(OH) | Mannans, glycogen |
| **1021** | **13.9** | **1021** | **8.8** | *δ*(CO) | Glucans |
| **992** | **3.4** | **988** | **1.7** | *ν*(CC), *δ*(CO) | *β*-1,6-Glucans |
| **964** | **1.0** | **966** | **2.1** | *ν*(CC), *δ*(CO) | Mannans, glycogen |
| **930** | **13.2** | **930** | **11.4** | *ν*(CC), *δ*(CO) | Mannans, glucans |
| **893** | **–** | **893** | **–** | *ν*(CC), *δ*(CO) | *β*-Glucan |
| **864** | **–** | **868** | **–** | *ν*(CC), *δ*(CO) | Glucan, mannan |
| **–** | **–** | **845** | **–** | *δ*(CH) | *α*-Configuration |
| **815** | **–** | **812** | **–** | *δ*(CH) | Glucan, mannan |
| **765** | **–** | **–** | **–** | *δ*(CH) | *α*-Glucan |
| **712** | **–** | **–** | **–** | *δ*(CH) | *α*-Glucan |

| **Pearson corellation coefficient, *r* =** | | | | **0.834** |  |
| --- | --- | --- | --- | --- | --- |
| ***r*^2^ =** | | | | **0.695** |  |
|  |  |  |  |  |  |
| **2918/2958** | **4.89** |  | **4.38** |  | Changes in lipid chain length, branching and/or saturation level |
| **2850/2873** | **0.38** |  | **0.05** |  |  |
| **AII/AI** | **0.47** |  | **0.26** |  | Changes in protein structure |
| **b-/a-Helix** | **12.6** |  | **NA** |  | Changes in secondary structure of proteins |
| **P/G** | **0.46** |  | **0.73** |  | Proteins (Amide I, II)/Glycans |
